# Characterization of Camphene- and Fenchol-Based Hydrophobic Eutectic Solvents and Their Application in Aldehyde Extraction

**DOI:** 10.3390/molecules29174232

**Published:** 2024-09-06

**Authors:** Alexander Kaufmann, Lars Häcker, Jacob Michael Mayer, Hansjörg Weber, Marlene Kienberger

**Affiliations:** 1 Institute of Chemical Engineering and Environmental Technology, Graz University of Technology, Inffeldgasse 25/C, 8010 Graz, Austria; alexander.kaufmann@tugraz.at (A.K.);; 2 Institute of Organic Chemistry, Graz University of Technology, Stremayergasse 9/A, 8010 Graz, Austria; hansjoerg.weber@tugraz.at

**Keywords:** eutectic solvents, solvent extraction, terpenes and terpenoids, vanillin, syringaldehyde, *p*-hydroxybenzaldehyde, solid–liquid equilibrium

## Abstract

Binary terpenoid-based eutectic systems consisting of the natural substances camphene (CA), fenchol (FE), thymol (TH), menthol (ME), dodecanoic acid (DA), and 1-dodecanol (DO) are synthesized and screened for their Solid–Liquid Equilibrium (SLE) and eutectic compositions. Out of nine eutectic systems, 13 solvent compositions at eutectic points and next to them, in addition to the reference solvent, TH:ME, are synthesized and applied for the solvent extraction of the aromatic aldehydes vanillin (VAN), syringaldehyde (SYR), and *p*-hydroxybenzaldehyde (HYD) from an acidic aqueous model solution. The extraction efficiency is determined from aldehyde concentrations measured by High-Performance Liquid Chromatography (HPLC), taking into consideration mutual solubility measured by Karl Fischer titration (KF) and a Total Organic Carbon-analysis (TOC). Physicochemical properties, such as the density, viscosity, and stability of the solvents, are evaluated and discussed. Additionally, ^1^H-NMR measurements are performed to verify hydrogen bonding present in some of the solvents. The results show that all synthesized eutectic systems have a strong hydrophobic character with a maximum water saturation of ≤2.21 vol.% and solvent losses of ≤0.12 vol.% per extraction step. The hydrophobic eutectic solvents based on CA exhibit lower viscosities, lower mutual solubility, and lower extraction efficiency for the aromatic aldehydes when compared with FE-based solvents. The highest extraction efficiencies for VAN (>95%) and for SYR (>93%) at an extraction efficiency of 92.61% for HYD are achieved by the reference solvent TH:ME (50:50 mol.%). With an extraction efficiency of 93.08%, HYD is most preferably extracted by the FE–DO-solvent (80:20 mol.%), where the extraction efficiencies for VAN and SYR reach their maximum at 93.37% and 90.75%, respectively. The drawbacks of the high viscosities of 34.741 mPas of the TH:ME solvent and 31.801 mPas of the FE–DO solvent can be overcome by the CA–TH solvent, which has a viscosity of 3.436 mPas, while exhibiting extraction efficiencies of 71.92% for HYD, >95% for VAN, and >93% for SYR, respectively.

## 1. Introduction

Solvent extraction is a core unit operation in many industrial processes and is used to extract valuable products from recyclable side streams, such as vanillin (VAN) and vanillic acid [1], or to remove harmful or toxic substances in wastewater treatment [2]. Due to their high extraction efficiency and good physicochemical properties, harsh and toxic solvents such as benzene [3] or chloroform [4], which are mainly produced using fossil resources rather than biorefineries [5], are often applied in solvent extraction processes. The worldwide CO_2_ emissions of 37.55 billion metric tons in 2023 [6] are causing negative effects on the environment and human health, increasing the need for improvements in industrial processes towards more sustainability. Therefore, the substitution of commonly applied industrial solvents with environmentally friendlier alternatives, following the principles of green chemistry [7], is an important contribution to reaching the UN’s sustainability goals, specifically in the area of “responsible consumption and production” [8].

Among green solvents, the group of neoteric solvents [9] consisting of Ionic Liquids (ILs), Deep Eutectic Solvents (DESs), supercritical fluids, and other biomass-derived solvents [10] plays an important role, exhibiting properties that are in accordance with the principles of green extraction [11]. ILs and DESs are typically binary mixtures consisting of either an anion and a cation or two compounds that establish strong molecular interactions, e.g., by hydrogen bonding [12]. These interactions can cause a melting point depression that enables a liquid aggregate state to be formed by solid educts at ambient conditions, such as in the case of the TH:ME-DES [13]. Furthermore, these mixtures can then be applied in solvent extraction using the synergy effects of the single components.

Whilst ILs often face drawbacks due to their complex synthesis routes and non-biodegradable raw materials, DESs can overcome those disadvantages at the cost of much higher viscosities [12]. Although DESs are often referred to as green solvents due to their bio-based origins, specific studies on their cytotoxicity and biodegradability are lacking. Two studies investigated the cytotoxicity of choline chloride-based DESs and phosphonium-based DESs on brine shrimps, where a higher cytotoxicity compared to their individual components was shown [14,15]. Therefore, the level of toxicity of a synthesized DES should always correlate with its specific use, for example, the further application of possible extracts from solvent extractions.

DESs are divided into types I to V, where type V consists of non-ionic species, and often exhibit hydrophobic characteristics and lower viscosities compared with types I to IV, which renders them ideal for use in solvent extractions from aqueous media [13,16]. The first hydrophobic DES (HDES) reported in the literature consisted of DL-menthol (DL-ME) with carboxylic acids [17] and combinations of decanoic acid and ammonium salts [18]. Currently, many publications, especially ones focusing on hydrophobic deep eutectic solvents, are available. The applications range from the extraction of various metal compounds [19,20,21] to applications as diluents or modifiers in reactive extractions [22,23] and as solvents for the extraction of dodecane [24] or pesticides [25] from aqueous phases or compounds, such as polyprenyl acetates from biomass [26]. In addition, the utilization of HDESs for CO_2_ capture was reported [27]. The main components used in those DESs consist of either ammonium salts or more natural substances like terpenes or terpenoids. Some advantages of using terpenes or terpenoids instead of ammonium salts can be seen in the comparison of decanoic acid-based HDESs, where HDESs utilizing terpenes as complementary compounds can exhibit lower viscosity and water uptake [18,28].

This special group of type V DESs consisting of mixtures of terpenes or terpenoids [29] has already been applied to the extraction of phenolic substances from wastewater and other aqueous solutions [2,30,31,32,33]. The thymol (TH)–menthol (ME) DES, a well-known representative of this type of DESs, exhibits strong deviations from the ideal Solid–Liquid Equilibrium (SLE) and is therefore used as a reference for the solvent extractions applied in this work [13,34,35,36].

Applications of HDESs as solvents for the extraction of the aromatic aldehydes VAN, SYR, and HYD are scarce. The only study found reports the extraction of VAN, caffeine, isophthalic acid, and tryptophan from aqueous media by applying DESs based on DL-ME [17].

The focus of this work lies on the synthesis of eutectic solvents based on the terpenoid fenchol (FE) and the terpene camphene (CA) in combinations with the terpenoids ME and TH as well as the long-chained fatty alcohol 1-dodecanol (DO) and the fatty acid dodecanoic acid (DA). Synthesized eutectic solvents based on FE have already been reported for the extraction of lutein from microalgae [37], quinines [38], or phenols [31] from aqueous solutions, for the solid–liquid extraction of artemisinin [39], and for special applications as part of a ferrofluid for fungicide analysis [40]. In addition, the application of FE combined with 10-undecenoic acid was used to extract ethanol, propan-1-ol, and propan-2-ol from water [41]. DESs based on CA have solely been characterized for their SLE and molecular interactions [42,43]. To the best of our knowledge, the systems CA–FE, CA–DA, FE–DA, and FE–DO have not been investigated for their SLE nor have they been applied as solvents for extraction purposes, which is presented in this paper.

Besides their strong hydrophobic characteristics and high stability during solvent extractions, the synthesis of sustainable HDESs is based on the origin and production routes of their raw materials. FE is a naturally occurring terpenoid that can be extracted from plants in addition to being produced by the biosynthesis of geranyl pyrophosphate [31,44]. CA is mainly produced by the reformation of the natural substance pinene over an acidic catalyst [45,46]. The terpenoids ME and TH are typically extracted from respective plants [47,48,49], but they can also be synthesized from other terpenes such as *m*-cresol [49,50]. DO and DA are typically produced from coconut or palm kernel oil [51,52], and biotechnological synthesis routes are also reported [53,54].

In this work, CA- and FE-based binary mixtures are investigated for the formation of a eutectic system, and they are characterized by the determination of their physicochemical properties and finally applied to the solvent extraction of the aromatic aldehydes VAN, SYR, and HYD from acidic aqueous media.

## 2. Results and Discussion

### 2.1. Synthesis of Eutectic Solvents and Appearance after 48 h

Table 1 summarizes the synthesized binary mixtures in this work. For simplification, these abbreviations are used throughout the entire work. Additional data on used components A and B are listed in Table S18 on page 13 in the Supplementary Materials.

**Figure 1:**
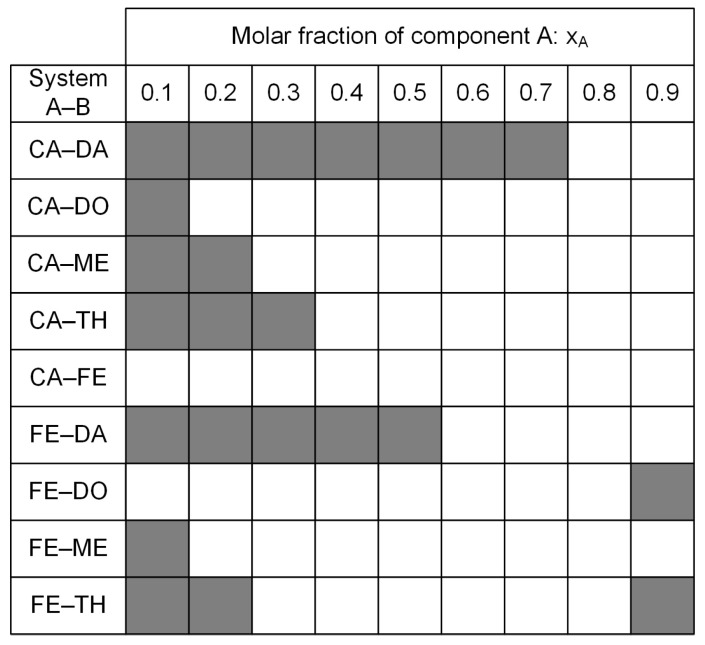
Aggregate state at ambient conditions after a minimum of 48 h after synthesis. Filled squares: solid/partially solid state; empty squares: liquid state.

Figure 1 shows the aggregate states of the eutectic mixtures prepared along the molar concentrations 48 h after synthesis and stored at ambient conditions. The filled squares represent the solid/partially solid state, and the empty squares represent a liquid aggregate state.

The systems CA–DA and FE–DA have a narrow range of the liquid aggregate state at ambient conditions towards higher DA concentrations, which can be attributed to the high melting temperature of 46 °C of DA. Compared with all other systems, CA–FE appears to stay completely liquid along the prepared molar concentrations. At molar compositions of 80% of CA and FE, respectively, all synthesized mixtures remain liquid at room temperature.

### 2.2. SLE and Eutectic Compositions

**Figure 2:**
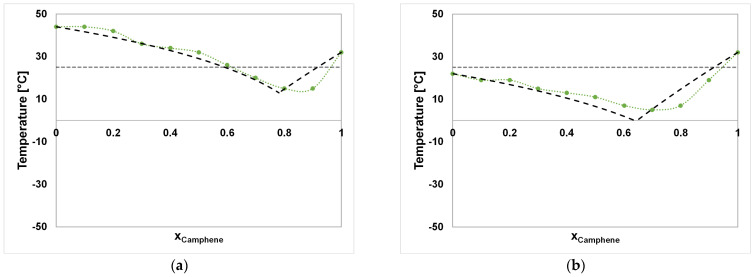

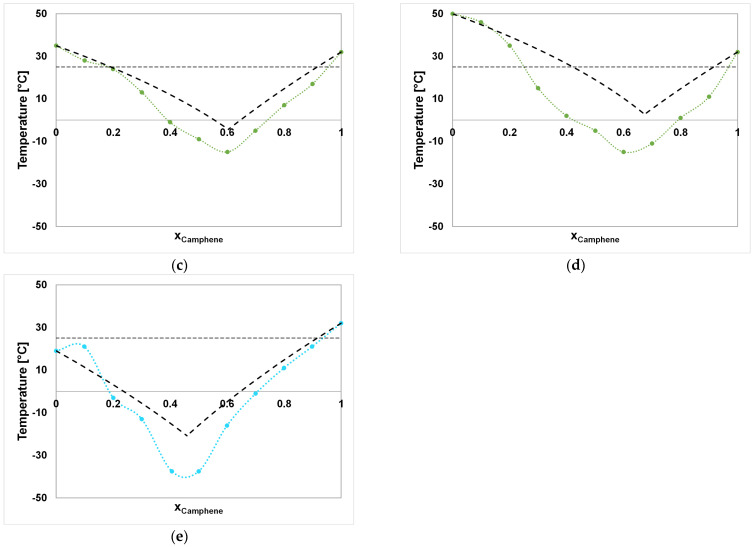
(**a**) CA–DA, (**b**) CA–DO, (**c**) CA–ME, (**d**) CA–TH, and (**e**) CA–FE. The black dashed lines show the ideal Solid–Liquid Equilibrium (SLE) according to Equation (1) on page 14. The green/light blue dotted lines are interpolations between the measured data points (green and light blue circles). The gray dashed lines represents 25 °C.

**Figure 3:**
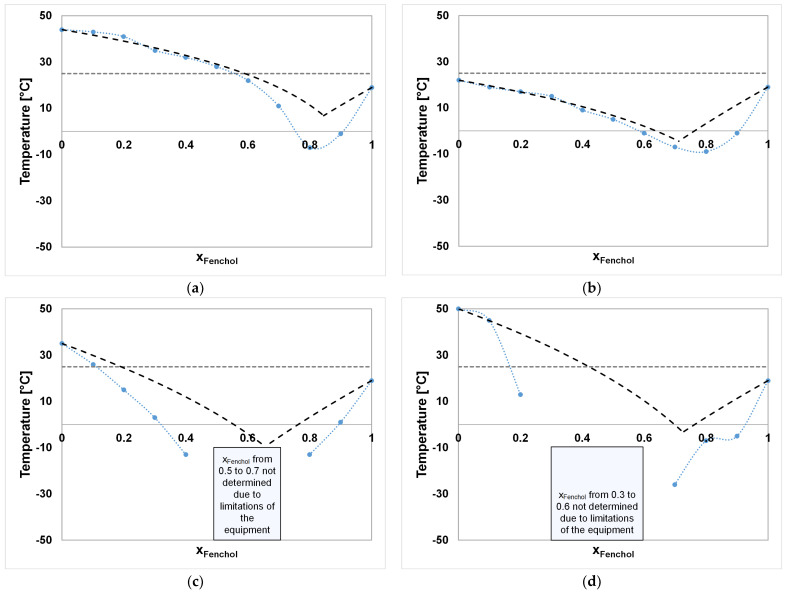
(**a**) FE–DA, (**b**) FE–DO, (**c**) FE–ME, and (**d**) FE–TH. The black dashed lines show the ideal SLE according to Equation (1) on page 14. The blue dotted lines are interpolations between the measured data points (blue circles). The gray dashed lines represents 25 °C.

In Figure 2a–e and Figure 3a–d, the experimentally determined SLEs of the investigated binary systems are plotted side by side with their ideal eutectic behaviors according to Equation (1). The ideal and experimental melting temperatures and eutectic compositions are stated in Table 2.

The CA–DA system (Figure 2a), shows a liquid aggregate state at 25 °C between a molar composition of >0.6 and 0.9 of CA, with only small deviations from the ideal behavior. The eutectic points deviate within 2 °C, resulting in eutectic compositions of 0.78 and 0.85 molar amounts of CA for the calculated and measured eutectic compositions, respectively. In addition, the systems CA–DA and CA–DO (Figure 2b) exhibit melting temperatures along the molar compositions that are solely above 0 °C. In contrast to the CA–DA system, the CA–DO system gives a nearly complete range of the liquid aggregate state at 25 °C, which makes it more versatile for applications in solvent extractions, where the properties might be adjusted by varying the composition. In addition, CA–DA and CA–DO are the only systems among those investigated where the calculated ideal behavior presents lower melting points than the ones determined experimentally. The SLE of the CA–DO system is already reported in the literature, where an experimental eutectic point was determined at approximately 0.6756 at a temperature of 283.6 K, fitting well to the values determined in this work [42]. The positive deviation from the ideal SLE was observed as well and addressed to possible packing effects of DO leading to less favorable van der Waals forces between the solvent components [42].

The systems CA–ME, CA–TH, and CA–FE represent eutectic behavior with a deviation of 10.6 °C to >16.69 °C from the calculated ideal melting points compared with the other CA-based mixtures. Here, CA–ME, CA–TH, and CA–FE show a liquid aggregate state from x_A_ > 0.2 to 0.9, from 0.3 to 0.9, and from 0 to 0.9 at 25 °C, respectively. The melting temperatures of CA and FE were measured as 32 °C and 19 °C, which happen to be much lower than those stated in the literature (T_m,CA_ = 52 °C, T_m,FE_ = 41.5 °C [55]). Using the values in the literature in the case of both components would result in much higher deviations from ideality but would not result in a significant change in the eutectic composition in the CA–FE system. The reason for the deviations of the pure component melting temperatures from the values in the literature may be attributed to the composition of the chemicals, which are often a mixture of different isomers, e.g., α-, and β-FE. In addition, the pure FE sample showed a melting temperature in the range of 19–21 °C in the SLE determinations while starting at a solid/partially liquid aggregate state at ambient conditions. The strong shift from ideality in the case of CA–FE, CA–ME, and CA–TH towards the eutectic composition might be attributed to the increasing amounts of intermolecular hydrogen bonds of FE, ME, and TH with a decreasing temperature, as in the case of other type V DESs such as TH–ME [34].

Negative deviations of 4.51 °C and 13.85 °C from the melting temperature of the ideal SLE are shown by the FE-based systems FE–DO and FE–DA. In both systems, hydrogen bonds between the two components of each mixture were detected by ^1^H-NMR measurements. The negative deviation of melting points in addition to the presence of hydrogen bonds between the single components identifies them as type V DESs [13]. The same applies to the systems FE–ME and FE–TH, for which the SLE is already described in the literature [37]. The SLE-curves of FE–ME and FE–TH were not fully determined along their molar compositions due to limitations of the cryostat with a minimal temperature of T_min_ = −37.5 °C, which could not denote the strong melting point depression in the ranges of x_A_ = 0.5 to 0.7 and x_A_ = 0.3 to 0.6, respectively. It is important to mention the visually inspected increase in viscosity, which made shaking impossible and might have led to a bias, especially for systems that inhabit strong melting point depressions such as FE–ME and FE–TH. In such cases, this type of melting point determination method might lead to inaccuracies. Therefore, the eutectic points for the systems FE–ME and FE–TH were not determined and thus were not further investigated.

According to the definition of a DES, the criteria of a liquid aggregate state at ambient operating conditions as well as a decrease in the melting temperature at the eutectic composition showing a deviation from an ideal eutectic mixture need to be fulfilled [56]. Therefore, CA–ME, CA–TH, and CA–FE, as well as FE–DA, FE–DO, FE–ME, and FE–TH, can be labeled as DESs, whilst CA–DA and CA–DO only represent simple eutectic mixtures. In any case, since the literature on the enthalpies of fusion is scarce and the ideal SLE was calculated by a group contribution method, obtaining a precise differentiation between deep eutectic and simple eutectic mixtures is rather difficult.

The masses for the synthesis of the mixtures prepared for the SLE determination and ideal SLE calculations are listed in Tables S1–S10 in the Supplementary Materials on pages 3–6.

Based on the solid–liquid curves, a mixture at the estimated eutectic composition, labeled as “composition A:composition B”, was synthesized for further investigations regarding physicochemical properties, stability, and suitability for the extraction of phenolic substances such as VAN, SYR, and HYD.

In addition, a mixture with a varying composition next to the eutectic point, labeled as “component A:component B *”, was synthesized to address the contribution and influence of the single components to the binary mixtures. Furthermore, the DES made from TH and ME (TH:ME) was synthesized and used as a reference eutectic solvent. Table 3 lists all eutectic solvents synthesized and investigated in this work. Due to the small area of a liquid aggregate state at 25 °C in the CA:DA system, no solvent at a composition next to the eutectic composition could be investigated.

### 2.3. Hydrogen Bond Determinations by Nuclear Magnetic Resonance Spectroscopy (NMR)

The formation of hydrogen bonds between the partners of a deep eutectic solvent can be monitored by measuring the ^1^H-NMR spectra of the individual components separately and in the DES mixture [37,38]. Typically, the OH-groups of the partners show a combined signal in the DES, suggesting hydrogen bonds. In addition, intermolecular Nuclear Overhauser Effects (NOEs) can sometimes be observed in the DES by the excitation of protons in close proximity to the hydrogen-bonding OH groups, which is a strong indication that the two partners are very close in space [23]. Both effects can be found in the ^1^H-NMR and NOESY1D spectra of the FE:DO and FE:DA DESs. These spectra and masses for the analyzed solvents can be found in Figures S1–S4 and in Table S23 on pages 17–19 in the Supplementary Materials.

### 2.4. Physicochemical Properties: Density and Viscosity

Based on similar studies of a TH:ME DES at molar compositions on both sides of the eutectic point, it can be assumed that physicochemical properties such as density and viscosity display linear behavior [57]. Therefore, the influence of a component can be directly attributed to its amount in the binary mixture. In Table 4, the densities and viscosities of the synthesized solvents are listed.

In the following section, all solvents are compared, where possible, based on the molar compositions.

In general, all synthesized CA-based solvents show lower dynamic viscosities in the range of 3 to 9 mPas compared with FE-based solvents, which show values between 21 and 35 mPas, similar to the reference solvent TH:ME with 34.741 mPas.

CA-based solvents show a decrease in density and viscosity with increasing amounts of CA. The addition of DO to CA:DO and ME to CA:ME solvents increases their viscosity with increasing amounts of DO and ME, respectively. Increasing amounts of TH and FE in CA:TH and CA:FE solvents results in decreases in viscosity and density. Comparing the influence of TH and FE on CA-based solvents at a similar molar composition leads to dynamic viscosities about 0.21 times higher in the case of FE at equivalent densities. Comparing the CA:ME with the CA:TH solvent shows a dynamic viscosity of 1.42 times that of CA:TH at a slightly decreasing density.

In contrast to CA-based solvents, FE-based solvents show an increase in density and viscosity with increasing amounts of FE.

Decreasing DA in FE:DA causes small increases in density and dynamic viscosity, with the latter being 1.09 times that of FE:DO at the same molar composition.

When DO is added to an equivalent molar composition to CA and FE, CA:DO solvents show 1.49 times lower viscosity compared with FE:DO solvents.

Comparing CA:FE with the reference solvent TH:ME, the dynamic viscosities are 3.10 times lower than the values of TH:ME, thereby overcoming one of the biggest disadvantages of hydrophobic eutectic solvents [12].

The dynamic viscosities of FE:DA and FE:DO exhibit comparable results to a reported solvent consisting of FE and α-terpineol in a ratio of 1:4, which shows 35.00 mPas at 298.15 K [39]. The dynamic viscosities of FE-based solvents are generally in the range of the TH:ME-DES, and can be even higher as shown in the cases of a FE:ME and FE:TH solvent, where dynamic viscosities of 32.90 mPas and 56.90 mPas at 298.15 K, respectively are reported [37].

The respective data for the triple determination of the density, dynamic viscosity, and kinematic viscosity measured are stated in Tables S19–S21 on pages 14–15 in the Supplementary Materials.

### 2.5. Extraction Efficiency for Aromatic Aldehydes

Table 5 shows the extraction efficiencies of the applied solvents for the extraction of the aromatic aldehydes HYD, VAN, and SYR.

Among all CA-based solvents, it can be seen that an increase in CA results in a decrease in the extraction efficiency for all aromatic aldehydes. Therefore, the increase in the complementary components DO, ME, TH, and FE to CA-based solvents increases the extraction efficiency for all three aldehydes. The extraction efficiency of CA:DA is significantly lower than that of all other synthesized solvents. Under the assumption that the main mechanism of aromatic aldehyde extraction with the proposed solvents is hydrogen bonding, as seen in a DES consisting of TH and VAN [58], the low extraction efficiency of CA:DA could be explained by it only consisting of 15% DA, which is responsible for possible hydrogen bond formation.

The specific influence on the extraction efficiency of TH, FE, and ME in CA-based solvents is shown by a preferred extraction of HYD when FE or ME is applied. To preferably extract VAN and SYR, the addition of TH to CA-based solvents is beneficial. When comparing the solvents with an equivalent molar composition of CA and a complementary component, such as CA:FE * with CA:TH * and CA:ME with CA:TH, the extraction efficiency for HYD increases by 12.86% and 12.23%, respectively. In the same comparison, the extraction efficiency for SYR increases by about >33.27% and >24.17%, showing the higher affinity of the complementary components FE and ME for the less polar aldehydes VAN and SYR.

The higher the proportion of FE in FE-based solvents, the higher the extraction efficiency for all three aldehydes. The addition of the complementary components DA and DO leads to a decrease in the extraction efficiency in FE-based solvents with increasing proportion. At equal molar compositions of DA and DO, the extraction efficiencies for all aldehydes are slightly higher when DO is added. For example, a 4.66% higher efficiency for HYD is achieved when comparing FE:DA with FE:DO.

The high affinity of DO for all three aldehydes can be seen by comparing FE:DO * and CA:DO *, where the differences in extraction efficiencies are only 2.69%, 3.54%, and 6.89% for HYD, VAN, and SYR, respectively.

The highest extraction efficiency for VAN with >95% and SYR with >93% is achieved by CA:TH and TH:ME, exceeding the limits of quantification of the High-Performance Liquid Chromatography–Ultra Violet (HPLC-UV) method. With an extraction efficiency of 93.08%, the FE:DO solvent achieves the best results for HYD extraction.

Taking the properties of mutual solubility and viscosity into account, CA:TH exhibits similar extraction efficiency compared with TH:ME at approximately one-tenth of dynamic viscosity.

Additional data, such as data on the masses of synthesized solvents, masses of feed and solvent for extractions, and respective HPLC data, are listed in Tables S11 and S14–S17 on pages 7, 10, and 11–12 in the Supplementary Materials.

### 2.6. Mutual Solubility

**Figure 4:**
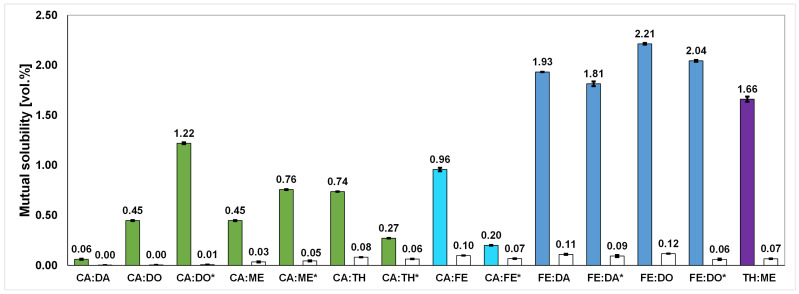
Mutual solubility from extraction experiments with an acidic aqueous solution. The colored/filled columns represent the water uptake in vol.% in the solvent measured by Karl Fischer titration (KF); the white/empty columns show the solvent losses into the resulting aqueous raffinate in vol.% determined by a Total Organic Carbon (TOC) analysis. Solvents marked with “*” represent compositions next to the eutectic point of the respective system.

The mutual solubility from contact with acidic aqueous media, as depicted in Figure 4, generally results in solvent losses of ≤0.12 vol.% and a water uptake of ≤2.21 vol.% for all synthesized solvents. This confirms their hydrophobic characteristics, hence confirming their beneficial properties for applications in liquid–liquid extractions.

An increase in CA in CA-based solvents goes hand in hand with a decrease in mutual solubility. The solvents CA:TH * and CA:FE * show similar mutual solubility at the same molar composition, while a comparison of CA:ME and CA:TH displays a 0.64 times higher water uptake in the case of CA:TH. A comparison with a HDES consisting of FE and 10-undecenoic acid shows water saturation between 15.648 and 20.864 ppm, which lies in a similar range to the proposed FE-based HDES [41]. In addition, the CA-based solvents proposed in this work exhibit lower water uptakes compared to that of solvents such as DL-ME and DA (2:1) proposed in the literature, which show a low water saturation of 1.237 wt.% [17].

The increase in FE in FE-based solvents generally results in an increase in mutual solubility. Here, FE:DO reaches the highest solvent loss with 2.21 vol.% and a water uptake of 0.12 vol.% among all synthesized solvents, which is still rather low compared with commonly applied solvents such as ethyl acetate [59]. Adding equivalent amounts of DA and DO to FE results in similar mutual solubility, with FE:DO having a 0.28 vol.% higher water uptake. A greater difference is observed when the equimolar addition of DO to FE and CA is compared. Here, FE:DO * has approximately 0.67 times more uptake of water.

The reference solvent TH:ME exhibits a mutual solubility between CA- and FE-based solvents, where a direct comparison with CA:FE shows that the water uptake of TH:ME is 1.73 times that of CA:FE.

The raw data for water uptakes and solvent losses are listed in Tables S12 and S13 on pages 8 and 9 in the Supplementary Materials.

### 2.7. Deviation in Solvent Composition (Stability) during Solvent Extraction

The composition of a binary solvent needs to remain constant during extraction to ensure consistent solvent properties, such as density, viscosity, a liquid aggregate state at operating conditions, and a sufficient extraction efficiency for the targeted solute. Table 6 lists the results of the Gas Chromatography (GC) measurements of the investigated solvents before and after extraction with the TOC model solution. The variation in the GC peak ratios A_A_:A_B_ indicates whether one of the two components of a solvent is favorably dissolved into the aqueous phase. In general, the lower the solvent loss into the aqueous phase per extraction step, the lower the impact on the solvent’s molar composition if one component is favorably dissolved.

CA:DA consists of 85% CA, with CA also being preferably extracted from the solvent. A variation in the peak ratios with a value of 2.47% is rather low and, in addition to its low mutual solubility, negligible. The solvents CA:DO and CA:DO * also show a low change in the solvent composition after extraction, with DO being preferably dissolved. This dissolution behavior increases with an increasing amount of DO in the solvent.

CA:ME exhibits a peak area deviation of 5.55% after extraction that does not significantly change when the amount of ME is increased as in the CA:ME * solvent. The distinct higher stability of CA:TH can be seen when compared with CA:ME, where the peak area deviations are <0.5% at the same molar composition of CA to the complementary component. In general, DO-containing solvents show lower variations in peak ratios and therefore higher stability during extractions compared with all other solvents. This characteristic is more dominant in FE-based solvents than in CA-based ones. The FE:DO solvent shows the lowest variations in peak ratios over a broad range of compositions, presenting similar stability to the reference solvent TH:ME.

Keeping in mind that none of the synthesized solvents reaches solvent losses of more than 0.12 vol.% per extraction step or peak area deviations with a maximum of 8.63%, there is no significant influence on the solvents’ compositions. Therefore, the investigated solvents are labeled as hydrophobic, stable, and suitable for extraction with the investigated acidic aqueous model solutions.

Additional data from the GC measurements are stated in Table S22 on page 16 in the Supplementary Materials.

## 3. Materials and Methods

### 3.1. Materials

The eutectic systems were synthesized from (+,−)-CA (≥95%, CAS 79-92-5, Carl Roth, Karlsruhe, Germany), FE (≥96%, CAS 1632-73-1, Sigma Aldrich, Darmstadt, Germany), TH (≥99%, CAS 89-83-8, Carl Roth, Karlsruhe, Germany), L-ME (≥99%, CAS 2216-51-5, Sigma Aldrich, Darmstadt, Germany), DA (≥97.5%, CAS 143-07-7, Sigma Aldrich, Darmstadt, Germany), and DO (≥97.5%, CAS 112-53-8, Sigma Aldrich, Darmstadt, Germany). All substances were used as received.

The model solution used for solvent extractions consisted of a 0.1 M NaOH solution (NaOH-pellets max.98%, CAS 1310-73-2, J.T. Baker, now Avantor, Radnor, PA, USA), H_2_SO_4_ (98% (97+%), CAS 7664-93-9, Chem-Lab, Zedelgem, Belgium), and the three aromatic aldehydes VAN (not less than 99.5%, CAS 121-33-5, Jiaxing Zhonghua Chemical Co., Ltd., Jiaxing City, China), SYR (≥98%, CAS 134-96-3, Sigma Aldrich, Darmstadt, Germany), and HYD (≥98%, CAS 123-08-0, Carl Roth, Karlsruhe, Germany).

For the HPLC-UV analysis, eluents consisting of 0.01 M of H_3_PO_4_ (85%, CAS 7664-38-2, VWR, now Avantor, Radnor, PA, USA) and methanol (≥99.9%, CAS 67-56-1, Honeywell, Morristown, NJ, USA) were used. Ethyl vanillin (≥98.5%, CAS 121-32-4, Sigma Aldrich, Darmstadt, Germany) was used as an internal standard for HPLC sample preparation.

For Karl Fischer (KF) titrations and Total Organic Carbon (TOC) analysis, standard laboratory chemicals were used (Hydranal™ Solvent, Hydranal^®^ Titrant 5, both Honeywell, Morristown, NJ, USA and O_2(g)_, 25 wt.% H_3_PO_4_, 1 M HCl and ultrapure water).

Samples for GC measurements were diluted with ethanol (≥99.8%, CAS 64-17-5, Honeywell, Morristown, NJ, USA) before analysis.

For the structural analysis by ^1^H-NMR, deuterochloroform CDCL_3_ (99.8 atom.% D, CAS 865-49-6, Deutero GmbH, Kastellaun, Germany) was used.

More data regarding the solvents and aromatic aldehydes used in this work are listed in Table S18 on page 13 in the Supplementary Materials.

### 3.2. Synthesis of Eutectic Mixtures

**Figure 5:**
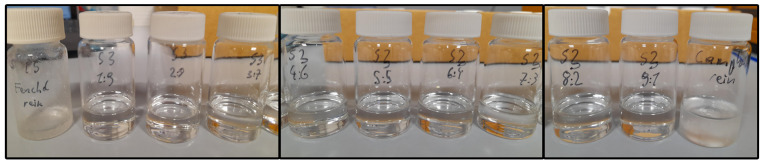
Mixtures of the system camphene–fenchol (CA–FE) along the molar compositions from left (pure FE) to right (pure CA) after a minimum of 48 h after synthesis.

Figure 5 shows an example of the binary mixtures from 0 to 100% of CA for the determination of the SLE, with the second compound being FE. The mixtures were prepared by weighing them to a total mass of 5 g in a glass vial while taking substance purities into account (5 g based on pure components). Synthesis was carried out by mixing the two components, followed by heating up the mixture above the melting temperature of the higher melting component until it was completely liquid, and finally subjecting it to further synthesis for a certain amount of time [23,33,60]. Therefore, the mixtures were put onto a heating plate, set to a temperature of 60–65 °C, and heated up until completely melted. Once melted, the mixtures were left at room temperature for 48 h, and the aggregate state was checked after every 24 h.

The eutectic systems applied in the solvent extractions were synthesized in a higher amount of 120 g total mass weighed into small Schott bottles supplied with a stirring bar and mounted in a water bath at 60–65 °C. When the mixture was completely liquid, which was visually observed, it was stirred for another 15 min at a constant temperature. The stirring bar was then removed, and the bottle was stored at ambient conditions and wrapped in alumina foil to avoid possible influences of sunlight. The aggregate state was again checked over 48 h. The solvents were synthesized at the respective eutectic compositions determined from SLE curves. Furthermore, solvents at compositions next to the eutectic point were prepared to investigate the influence of a single component on the solvent characteristics. In total, 14 solvents were synthesized, and their physicochemical properties together with characteristics such as stability, mutual solubility, and extraction efficiency for aromatic aldehydes during solvent extraction were investigated.

### 3.3. Determination of SLE

**Figure 6:**
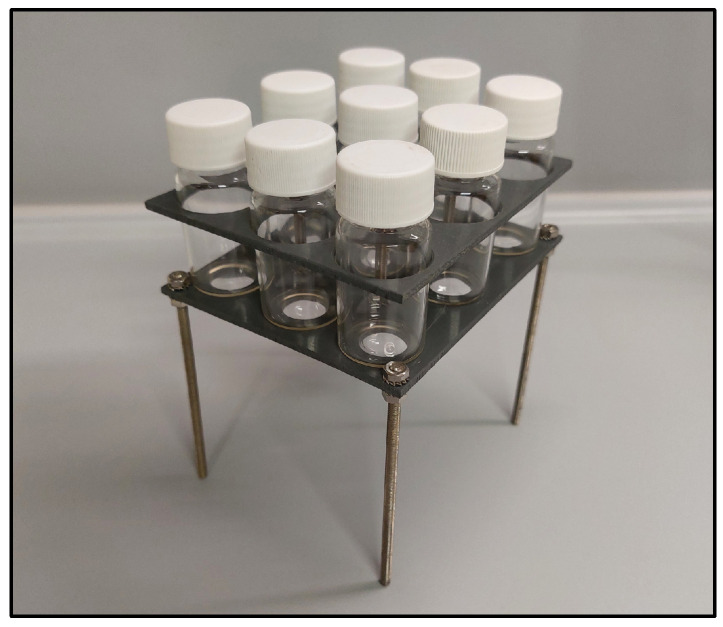
Sample rack used for SLE experiments, which was mounted in cryostat.

To obtain the SLE curves, a small stirring bar was added to the prepared 5 g mixtures, which were stacked into a rack, as shown in Figure 6, and mounted in the circulating bath of a Lauda ecoline RE 104 cryostat (Lauda—Königshofen, Germany).

The following procedure was applied to all samples: The starting temperature was set so that all mixtures that were solid at ambient conditions were liquid again. Therefore, the starting temperature varied depending on the mixtures investigated and was oriented to the values in the literature for the melting temperatures of the pure components listed in Table S10 on page 6 in the Supplementary Materials. Once this was ensured, the set temperature was decreased by 2 °C and kept constant for 15 min to ensure the complete equilibration of the mixtures. Then, the mixtures were taken out and thoroughly shaken to introduce mechanical energy to avoid a melting point depression caused by the undercooling of liquids. This procedure was repeated until all samples were solid again. In the case of melting temperatures below −20 °C, a Lauda ECO RE 1050 S (Lauda—Königshofen, Germany) was used, where a temperature of −37 °C was achievable. The determination of the SLE was carried out by single determination and in temperature increments of 2 °C, where the value of a solid aggregate state (low end value) was taken as the melting = solidification temperature.

The ideal SLE lines of a eutectic solution can be calculated using Equation (1), where the activity coefficient $\gamma_{i}^{L}$ is set to 1, assuming infinite dilution and transition terms are neglected for simplification [61].

The melting temperatures of the pure components $T_{m,i}$ were determined experimentally, while the molar enthalpies of fusion $∆h_{m,i}$ were estimated following the method used by Joback and Reid [62].

These curves were plotted in addition to the experimental results to compare deviations from the ideal system.

(1) $$\ln x_{i}^{L}\times \gamma_{i}^{L}=-\frac{∆h_{m,i}}{RT}\times \left(1-\frac{T}{T_{m,i}}\right)$$

### 3.4. Nuclear Magnetic Resonance Spectroscopy (NMR)

NMR measurements were carried out on a Jeol JNM-ECZL 500 MHz spectrometer (Tokyo, Japan) at 30 or 35 °C using the Delta 6.1 software. ^1^H-NMR spectra were measured in solutions of CDCl_3_ at 499.7189 MHz on a Royal HFX-Probe (Jeol, Tokyo, Japan), (Automatic Tuning and Matching). ^1^H-NMR parameters were as follows: relaxation delay of 2 s; 45° proton pulse; acquisition time of 2.048 s; spectral width of 8 kHz; and 32 k points. Sixteen scans were accumulated. NOESY1D is an excitation-sculpted transient NOE experiment with selective excitation of user-defined signals by shaped pulses (iburp) in a double pulsed field gradient spin echo. Mixing time was 500 ms unless otherwise stated. Typically, 128 to 1024 scans were accumulated.

### 3.5. Physicochemical Properties: Density and Viscosity

The density and viscosity of all 14 solvents were measured with an Anton Paar SVM 3000 (Graz, Austria) at a temperature of 25 °C. Measurements were performed in triplicate.

### 3.6. Solvent Extraction and Mutual Solubility

The solvent extractions were carried out by mixing 20 mL of model solution and 20 mL of solvent into a Janke & Kunkel IKA-Werk HS500 (Staufen, Germany) linear oscillating laboratory shaker equipped with a Lauda ecoline RE104/E100 (Lauda—Königshofen, Germany) for constant temperature control at 25 °C. The procedure consisted of 1 h of shaking at 180 strokes/min followed by settling for complete phase separation over 5 h.

Total Organic Carbon extractions (TOC extractions) with the model solutions without aldehydes (TOC model solution) were also carried out at an S:F-ratio of 1:1 and at 25 °C, applying the same extraction procedure.

Mutual solubility was accounted for by calculating the amount of water uptake measured by KF with a Titroline^®^ 7500KF automatic titrator from SI Analytics (Mainz, Germany, now Xylem Inc., Washington, DC, USA) and by calculating the amount of solvent loss into the raffinate measured byTOC with a Shimadzu TOC-L (Kyoto, Japan). For the TOC-analysis, it was assumed that dissolution into the aqueous raffinate is non-selective for the two components from which the eutectic solvent is synthesized.

**Figure 7:**
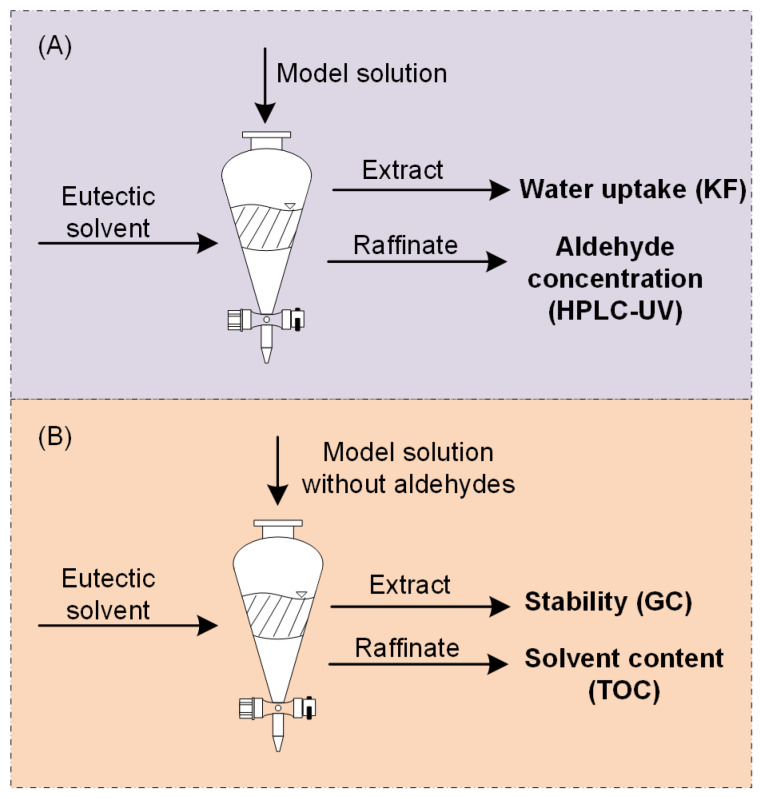
(**A**) Extractions with model solutions for the analysis of water uptake and aldehyde concentrations. (**B**) Extractions with a model solution without aldehydes (TOC extractions) for solvent loss and stability measurements. Figure adapted from [63].

Aldehyde concentrations in the raffinate phases were determined by HPLC-UV according to Figure 7. Measurements of water uptake were carried out in triplicate, and analyses of solvent loss and aldehyde concentrations were carried out in duplicate.

### 3.7. Determination of Extraction Efficiency with HPLC-UV

For the calculation of extraction efficiencies, aldehyde concentrations were measured with a Shimadzu UFLC Prominence HPLC (Kyoto, Japan) equipped with a UV/VIS detector at 254 nm. The column used was a Phenomenex^®^ Gemini 5 µ C18 110A (Phenomenex, now Danaher, Washington, DC, USA) with a size of 150 × 4.60 mm at a constant column oven temperature of 30 °C. The method applied was an isocratic flow at 0.7 mL min^−1^, with the mobile phase consisting of 0.01 M phosphoric acid and methanol in a ratio of vol.%:vol.% = 82:18. The total method’s runtime was 45 min with approximate retention times for the investigated aromatic aldehydes of 13.1 min for HYD, 17.25 min for VAN, and 21.7 min for SYR. For an accurate evaluation, ethyl vanillin was used as an internal standard (retention time: 32.3 min) to correct the measured aldehyde peaks. The sample preparation procedure consisted of the addition of 1 mL of internal standard and 3 mL of methanol to 1 mL of sample before filtering it through 0.45 µm PES filters into a HPLC vial sealed with a cap. The method and existing calibrations for the internal standard and analytes are already reported in [63].

The extraction efficiency EE according to Equation (3) is calculated from the masses of aldehydes $m_{aldehyde,i}$ in the feed and the raffinate phases, where the index i labels the respective phase with *F* for the feed and *R* for the raffinate. The masses are calculated according to Equation (2) with aldehyde concentrations $\beta_{aldehyde,i}$ from HPLC-UV measurements and the respective volumes $V_{i}$, which were determined by volume balances while considering mutual solubility. The procedure for the volume balances, taking mutual solubility into account, is described in [63].

(2) $$m_{aldehyde,i}=\beta_{aldehyde,i}\times V_{i}\;\left[g\right]$$

(3) $$EE=\left(1-\frac{m_{aldehyde,R}}{m_{aldehyde,F}}\right)\times 100\;\left[\%\right]$$

### 3.8. Stability Analysis by GC

The stability of a solvent was evaluated by analyzing the ratio of component A to B of the synthesized solvent before and after TOC extractions using GC. To determine whether a solvent dissolves in the same molar composition or if one component is extracted to a higher extent, the relative deviations of the peak area ratio of component A to component B of the eutectic solvent before and after extraction with the TOC model solution is compared. The system used was an Agilent Technologies 6890N Network GC System (Santa Clara, CA, USA) equipped with an FID detector at 300 °C and a DB-624 UI column with dimensions of 30 m × 0.250 mm with 1.40 microns. The solvents and extracts were diluted with ethanol into a range operatable for the detector, and the following method was applied: T_start_ = 50 °C (hold for 1 min), followed by heating to 250 °C with a rate of 10 °C min^−1^, and then keeping that temperature constant for another 20 min.

## 4. Conclusions

The combination of the natural substances CA, FE, TH, ME, DA, and DO was utilized for the synthesis of nine hydrophobic eutectic systems, where six of them were reported for the first time. The binary mixtures were characterized by the determination of their SLEs, eutectic compositions, and physicochemical properties such as density and viscosity. From the synthesized eutectic systems, 13 solvents were synthesized at the eutectic composition and next to it. Those solvents, in addition to the reference solvent TH:ME, were investigated for extraction efficiency, mutual solubility, and stability during the solvent extraction of the aromatic aldehydes VAN, SYR, and HYD from an acidic aqueous model solution.

The systems FE–DO, FE–DA, FE–ME, FE–TH, CA–ME, CA–TH, and CA–FE showed negative deviations from ideal eutectic behavior and were therefore labeled as DESs. The presence of hydrogen bonds in the solvents FE:DA and FE:DO was determined by ^1^H-NMR. The CA–DA and CA–DO systems did not exhibit the mentioned non-ideality and were therefore characterized as simple eutectic solvents.

All synthesized solvents exhibited low mutual solubility, with solvent losses of ≤0.12 vol.% and water uptakes of ≤2.21 vol.% during extractions at 25 °C. CA-based solvents were characterized by a lower mutual solubility, lower viscosities, and lower extraction efficiencies compared with the FE-based solvents.

None of the synthesized solvents exceeded the extraction efficiencies of the reference solvent TH:ME for VAN and SYR with values of >95% and >93%, respectively. The highest extraction efficiency for HYD was achieved by the FE:DO solvent with 93.08%. Taking physicochemical properties and mutual solubility into account, the CA:TH solvent reached extraction efficiencies of 71.92% for HYD, >95% for VAN, and >93% for SYR at a solvent loss of 0.08 vol.% and a water uptake of 0.74 vol.%. Based on the solvents of the CA–TH-system, a shift to a composition of, e.g., CA:TH = 0.30:0.70, might improve the extraction efficiency for HYD while still keeping the density, viscosity, and mutual solubility below the properties of the reference solvent TH:ME.

## Figures and Tables

**Table 1:** Abbreviations for the eutectic systems. The eutectic system is always written in the form of Component A–Component B.

System A–B	Component A	Component B
CA–DA	Camphene (CA)	Dodecanoic Acid (DA)
CA–DO	Camphene (CA)	1-Dodecanol (DO)
CA–ME	Camphene (CA)	L-Menthol (ME)
CA–TH	Camphene (CA)	Thymol (TH)
CA–FE	Camphene (CA)	Fenchol (FE)
FE–DA	Fenchol (FE)	Dodecanoic Acid (DA)
FE–DO	Fenchol (FE)	1-Dodecanol (DO)
FE–ME	Fenchol (FE)	L-Menthol (ME)
FE–TH	Fenchol (FE)	Thymol (TH)

**Table 2:** Experimentally determined and calculated ideal eutectic compositions and melting temperatures of the investigated binary systems. The eutectic compositions and melting temperatures of the systems FE–ME and FE–TH have not been determined (marked with n.a.) due to their strong melting point depression and the limits of the used cryostat.

System A–B	Ideal SLE x_A_ [ ]	Ideal SLE T_m_ [°C]	Experimental SLE x_A_ [ ]	Experimental SLE T_m_ [°C]
CA–DA	0.78	13.00	0.85	≤15
CA–DO	0.64	−0.33	0.70	≤7
CA–ME	0.60	−4.40	0.60	−15
CA–TH	0.67	2.73	0.60	−15
CA–FE	0.46	−20.81	0.45	≤−37.5
FE–DA	0.84	6.85	0.80	−7
FE–DO	0.71	−4.49	0.80	−9
FE–ME	0.66	−9.52	n.a.	n.a.
FE–TH	0.73	−3.28	n.a.	n.a.

n.a.—not available.

**Table 3:** Solvents synthesized at eutectic compositions approximated from the curve fit, the TH:ME from the literature, and the solvent * synthesized next to the eutectic point.

Solvent A:B	Composition x_A_:x_B_	Solvent *	Composition x_A_:x_B_
CA:DA	0.85:0.15	n.a.	n.a.
CA:DO	0.70:0.30	CA:DO *	0.40:0.60
CA:ME	0.60:0.40	CA:ME *	0.42:0.58
CA:TH	0.60:0.40	CA:TH *	0.80:0.20
CA:FE	0.44:0.56	CA:FE *	0.80:0.20
FE:DA	0.80:0.20	FE:DA *	0.70:0.30
FE:DO	0.80:0.20	FE:DO *	0.40:0.60
TH:ME	0.50:0.50 [36]	n.a.	n.a.

“n.a.”—not available.

**Table 4:** Physicochemical properties, including density (ρ), dynamic viscosity (η), and kinematic viscosity (ν), of synthesized solvents measured at 25 °C. The solvents marked with “*” refer to the compositions next to the eutectic point.

Solvent	x_A_:x_B_	ρ [g cm^−3^]	η [mPas]	ν [mm^2^ s^−1^]
CA:DA	0.85:0.15	0.868 ± 0.000	3.136 ± 0.006	3.612 ± 0.006
CA:DO	0.70:0.30	0.850 ± 0.001	4.454 ± 0.023	5.238 ± 0.025
CA:DO *	0.40:0.60	0.840 ± 0.001	8.476 ± 0.035	10.085 ± 0.039
CA:ME	0.60:0.40	0.876 ± 0.000	4.894 ± 0.011	5.585 ± 0.011
CA:ME *	0.42:0.58	0.881 ± 0.000	8.944 ± 0.074	10.147 ± 0.083
CA:TH	0.60:0.40	0.905 ± 0.000	3.436 ± 0.002	3.796 ± 0.002
CA:TH *	0.80:0.20	0.884 ± 0.001	2.526 ± 0.011	2.867 ± 0.020
CA:FE	0.44:0.56	0.913 ± 0.000	8.465 ± 0.068	9.269 ± 0.074
CA:FE *	0.80:0.20	0.882 ± 0.000	3.067 ± 0.019	3.479 ± 0.022
FE:DA	0.80:0.20	0.937 ± 0.001	34.617 ± 0.813	36.944 ± 0.846
FE:DA *	0.70:0.30	0.929 ± 0.000	29.514 ± 0.396	31.765 ± 0.428
FE:DO	0.80:0.20	0.923 ± 0.001	31.801 ± 0.532	34.466 ± 0.551
FE:DO *	0.40:0.60	0.871 ± 0.000	21.131 ± 0.124	24.257 ± 0.135
TH:ME	0.50:0.50	0.933 ± 0.000	34.741 ± 0.32	37.252 ± 0.343

**Table 5:** Extraction efficiencies (EEs) of synthesized solvents for the extraction of the aromatic aldehydes *p*-hydroxybenzaldehyde (HYD), vanillin (VAN), and syringaldehyde (SYR) from a 1 g·L^−1^ model solution at 25 °C. Solvents marked with a “*” represent compositions next to the eutectic composition.

Solvent	x_A_:x_B_	EE_HYD [%]	EE_VAN [%]	EE_SYR [%]
CA:DA	0.85:0.15	8.43 ± 0.13	44.59 ± 0.36	26.50 ± 0.21
CA:DO	0.70:0.30	81.48 ± 0.32	79.51 ± 0.35	66.90 ± 0.64
CA:DO *	0.40:0.60	89.80 ± 0.29	87.81 ± 0.63	80.03 ± 1.44
CA:ME	0.60:0.40	84.15 ± 0.50	81.26 ± 0.67	68.83 ± 1.23
CA:ME *	0.42:0.58	90.07 ± 0.19	87.14 ± 0.32	77.75 ± 0.73
CA:TH	0.60:0.40	71.92 ± 0.26	>95.00	>93.00
CA:TH *	0.80:0.20	42.69 ± 0.49	90.50 ± 0.11	>93.00
CA:FE	0.44:0.56	85.85 ± 0.40	89.09 ± 0.31	84.60 ± 0.21
CA:FE *	0.80:0.20	55.55 ± 1.12	71.77 ± 0.85	59.73 ± 0.00
FE:DA	0.80:0.20	88.42 ± 0.12	90.71 ± 0.01	87.25 ± 0.41
FE:DA *	0.70:0.30	84.86 ± 0.19	88.31 ± 0.38	85.03 ± 0.79
FE:DO	0.80:0.20	93.08 ± 0.05	93.37 ± 0.10	90.75 ± 0.32
FE:DO *	0.40:0.60	92.49 ± 0.14	91.35 ± 0.15	86.92 ± 0.07
TH:ME	0.50:0.50	92.61 ± 0.16	>95.00	>93.00

**Table 6:** Deviations of peak ratios in Gas Chromatography analysis (GC) before and after extraction with the TOC model solution. First named component (A); second named component (B) (e.g., CA:DA = A:B). For deviations < 0.5, no preferably dissolved component is stated, marked with “-”. Solvents marked with a “*” represent compositions next to the eutectic composition.

Solvent A:B	x_A_:x_B_	A_A_:A_B_ before Extraction	A_A_:A_B_ after Extraction	Absolute Deviation [%]	Preferably Dissolved Component:
CA:DA	0.85:0.15	14.66	14.30	2.47	CA
CA:DO	0.70:0.30	2.75	2.78	<1	DO
CA:DO *	0.40:0.60	0.79	0.80	1.67	DO
CA:ME	0.60:0.40	1.79	1.89	5.55	ME
CA:ME *	0.42:0.58	0.76	0.80	5.49	ME
CA:TH	0.60:0.40	1.80	1.80	<0.5	-
CA:TH *	0.80:0.20	5.27	5.16	2.07	CA
CA:FE	0.44:0.56	0.99	1.01	2.19	FE
CA:FE *	0.80:0.20	5.19	5.22	<1	FE
FE:DA	0.80:0.20	6.91	6.57	4.91	FE
FE:DA *	0.70:0.30	3.83	3.50	8.63	FE
FE:DO	0.80:0.20	4.28	4.28	<0.5	-
FE:DO *	0.40:0.60	0.70	0.70	<0.5	-
TH:ME	0.50:0.50	0.99	0.99	<0.5	-

## Data Availability

All data are published in the manuscript and the Supplementary Materials.

## Supplementary Materials

The following Supplementary Materials can be downloaded at https://www.mdpi.com/article/10.3390/molecules29174232/s1, Tables S1–S9: Masses of components for the mixtures used in SLE determination; Table S10: Data for ideal SLE calculation; Table S11: Masses of synthesized solvents applied in solvent extractions; Table S12: Water uptake of solvents during solvent extraction; Table S13: Solvent losses into aqueous phases during solvent extraction; Table S14: Masses of feed and model solution for solvent extractions; Table S15: HPLC data of measured raffinate phases. Table S16: HPLC data of measured model solutions (feed of solvent extraction); Table S17: Calibration data of HPLC; Table S18: Purity, molecular weights, and structures of used chemicals for eutectic systems and aromatic aldehydes; Tables S19–S21: Density and viscosity data from measurements made with Anton Paar SVM 3000; Table S22: GC areas of diluted solvents before and after TOC extractions; Table S23: Masses of FE:DA and FE:DO analyzed by ^1^H-NMR; Figures S1–S4: Spectra from ^1^H-NMR measurements.
