# Supplementary material for: Characterization of Camphene- and Fenchol-Based Hydrophobic Eutectic Solvents and Their Application in Aldehyde Extraction
Source: Molecules. 2024 Sep 6;29(17):4232. doi: 10.3390/molecules29174232 (PMC11397249; doi:10.3390/molecules29174232)
Supplement: Supplementary file 1 [file molecules-29-04232-s001.zip › molecules-3081153-supplementary.pdf]

Supplementary Materials

Characterization of Camphene- and Fenchol-Based Hydrophobic Eutectic Solvents and their Application in Aldehyde Extraction

Alexander Kaufmann[1], Lars Häcker[1], Jacob Michael Mayer[1], Hansjörg Weber[2], Marlene Kienberger[1,]*

[1]Institute of Chemical Engineering and Environmental Technology, Graz University of Technology, Inffeldgasse 25/C, 8010 Graz; Austria

[2]Institute of Organic Chemistry, Graz University of Technology, Stremayergasse 9/A, 8010 Graz; Austria

alexander.kaufmann@tugraz.at (Alexander Kaufmann)
larshaecker@gmx.de (Lars Häcker)
jacob.mayer@student.tugraz.at (Jacob Michael Mayer)
hansjoerg.weber@tugraz.at (Hansjörg Weber)
marlene.kienberger@tugraz.at* (Marlene Kienberger)

Content

1 Data for Solid-Liquid Equilibrium (SLE) 2
1.1 Calculation for SLE 2
1.2 Solvent synthesis data for SLE determinations 3
1.3 Data for Ideal SLE calculations 6
2 Data for solvent extraction of aldehydes 7
2.1 Solvent synthesis data for solvent extraction 7
2.2 Water uptake during solvent extraction 8
2.3 Solvent loss during solvent extraction 9
2.4 Masses of feed and solvent for solvent extractions 10
2.5 HPLC-UV data 11
3 Material-and solvent-properties 13
4 GC-Data 16
5 ^{1}H-NMR spectra of FE:DO and FE:DA 17
6 References 20

1 Data for Solid-Liquid Equilibrium (SLE)

1.1 Calculation for SLE

Following calculations are also valid for the synthesis of the solvents applied in the solvent extractions and ^{1}H-NMR analysis.

1) Determination of the needed masses of components A and B:

$$m_{A,needed} = \frac{m_{total} * x_{A,needed} * Mw_A}{\left(Mw_A * x_{A,needed} + Mw_B * x_{B,needed}\right) * Purity_A} \qquad [g] \qquad \text{Eq S1}$$

$$m_{B,needed} = \frac{m_{total} * x_{B,needed} * Mw_B}{\left(Mw_A * x_{A,needed} + Mw_B * x_{B,needed}\right) * Purity_B} \qquad [g] \qquad \text{Eq S2}$$

2) Calculation of the actual molar amounts of components A and B:

$$n_{Comp.A} = \frac{m_{Comp.A} * Purity_A}{Mw_A} \qquad [mol] \qquad \text{Eq S3}$$

$$n_{Comp.B} = \frac{m_{Comp.B} * Purity_B}{Mw_B} \qquad [mol] \qquad \text{Eq S4}$$

3) Calculation of the actual molar fractions of components A and B:

$$x_{Comp.A} = \frac{n_{Comp.A}}{\left(n_{Comp.A} + n_{Comp.B}\right)} \qquad \left[\frac{mol}{mol}\right] \qquad \text{Eq S5}$$

$$x_{Comp.B} = \frac{n_{Comp.B}}{\left(n_{Comp.A} + n_{Comp.B}\right)} \qquad \left[\frac{mol}{mol}\right] \qquad \text{Eq S6}$$

1.2 Solvent synthesis data for SLE determinations

Table S1. Weighed masses and calculated molar compositions of components A and B and determined solidification temperature for the system CA–DA.

$m_{Comp.A}$ $[g]$	$m_{Comp.B}$ $[g]$	$x_{Comp.A}$ $\left[\frac{mol}{mol}\right]$	$x_{Comp.B}$ $\left[\frac{mol}{mol}\right]$	$T_{solidification}$ $[°C]$
0.3661	4.7429	0.10	0.90	44
0.7655	4.3599	0.20	0.80	42
1.1909	3.9568	0.30	0.70	36
1.6499	3.5133	0.40	0.60	34
2.1275	3.0302	0.50	0.50	32
2.6543	2.5265	0.60	0.40	26
3.2232	1.9793	0.70	0.30	20
3.8574	1.3741	0.80	0.20	15
4.5299	0.7192	0.90	0.10	15

Table S2. Weighed masses and calculated molar compositions of components A and B and determined solidification temperature for the system CA–DO.

$m_{Comp.A}$ $[g]$	$m_{Comp.B}$ $[g]$	$x_{Comp.A}$ $\left[\frac{mol}{mol}\right]$	$x_{Comp.B}$ $\left[\frac{mol}{mol}\right]$	$T_{solidification}$ $[°C]$
0.3943	4.7192	0.10	0.90	19
0.8191	4.3312	0.20	0.80	19
1.2574	3.9003	0.30	0.70	15
1.7285	3.4388	0.40	0.60	13
2.2282	2.9543	0.50	0.50	11
2.7849	2.4413	0.60	0.40	7
3.3119	1.8865	0.70	0.30	5
3.9200	1.3051	0.80	0.20	7
4.5649	0.6823	0.90	0.10	19

Table S3. Weighed masses and calculated molar compositions of components A and B and determined solidification temperature for the system CA–ME.

$m_{Comp.A}$ $[g]$	$m_{Comp.B}$ $[g]$	$x_{Comp.A}$ $\left[\frac{mol}{mol}\right]$	$x_{Comp.B}$ $\left[\frac{mol}{mol}\right]$	$T_{solidification}$ $[°C]$
0.4656	4.6071	0.10	0.90	28
0.9378	4.1406	0.20	0.80	24
1.4360	3.6739	0.30	0.70	13
1.9376	3.2060	0.40	0.60	-1
2.4560	2.6986	0.50	0.50	-9
2.9907	2.1847	0.60	0.40	-15
3.5238	1.6700	0.70	0.30	-5
4.0995	1.1274	0.80	0.20	7
4.6809	0.5752	0.90	0.10	17

Table S4. Weighed masses and calculated molar compositions of components A and B and determined solidification temperature for the system CA–TH.

$m_{Comp.A}$ $[g]$	$m_{Comp.B}$ $[g]$	$x_{Comp.A}$ $\left[\frac{mol}{mol}\right]$	$x_{Comp.B}$ $\left[\frac{mol}{mol}\right]$	$T_{solidification}$ $[°C]$
0.4815	4.5892	0.10	0.90	46
0.9836	4.1398	0.20	0.80	35
1.4733	3.5620	0.30	0.70	15
1.9881	3.1353	0.40	0.60	2
2.5096	2.6507	0.50	0.50	-5
3.0347	2.1428	0.60	0.40	-15
3.5727	1.6209	0.70	0.30	-11
4.1262	1.0931	0.80	0.20	1
4.6988	0.5546	0.90	0.10	11

Table S5. Weighed masses and calculated molar compositions of components A and B and determined solidification temperature for the system CA–FE.

$m_{Comp.A}$ $[g]$	$m_{Comp.B}$ $[g]$	$x_{Comp.A}$ $\left[\frac{mol}{mol}\right]$	$x_{Comp.B}$ $\left[\frac{mol}{mol}\right]$	$T_{solidification}$ $[°C]$
0.5122	5.2209	0.10	0.90	21
0.9570	4.2713	0.20	0.80	-3
1.4501	3.7812	0.30	0.70	-13
2.0304	3.3265	0.41	0.59	-37.5
2.4714	2.7654	0.50	0.50	-37.5
3.0019	2.2454	0.60	0.40	-16
3.5548	1.7087	0.70	0.30	-1
4.1115	1.1546	0.80	0.20	11
4.6952	0.5962	0.90	0.10	21

Table S6. Weighed masses and calculated molar compositions of components A and B and determined solidification temperature for the system FE–DA.

$m_{Comp.A}$ $[g]$	$m_{Comp.B}$ $[g]$	$x_{Comp.A}$ $\left[\frac{mol}{mol}\right]$	$x_{Comp.B}$ $\left[\frac{mol}{mol}\right]$	$T_{solidification}$ $[°C]$
0.4181	4.6961	0.10	0.90	43
0.8387	4.2817	0.20	0.80	41
1.2995	3.8415	0.30	0.70	35
1.7645	3.3698	0.40	0.60	32
2.2620	2.8841	0.50	0.50	28
2.7975	2.3631	0.60	0.40	22
3.3412	1.8280	0.70	0.30	11
3.9329	1.2507	0.80	0.20	-7
4.5583	0.6480	0.90	0.10	-1

Table S7. Weighed masses and calculated molar compositions of components A and B and determined solidification temperature for the system FE–DO.

$m_{Comp.A}$ $[g]$	$m_{Comp.B}$ $[g]$	$x_{Comp.A}$ $\left[\frac{mol}{mol}\right]$	$x_{Comp.B}$ $\left[\frac{mol}{mol}\right]$	$T_{solidification}$ $[°C]$
0.4342	4.6720	0.10	0.90	19
0.8939	4.2390	0.20	0.80	17
1.3658	3.7686	0.30	0.70	15
1.8557	3.2880	0.40	0.60	9
2.3563	2.7989	0.50	0.50	5
2.8823	2.2791	0.60	0.40	-1
3.4254	1.7459	0.70	0.30	-7
4.0087	1.1840	0.80	0.20	-9
4.5915	0.6020	0.90	0.10	-1

Table S8. Weighed masses and calculated molar compositions of components A and B and determined solidification temperature for the system FE–ME. Solidification temperatures not determined are marked with "n.a.".

$m_{Comp.A}$ $[g]$	$m_{Comp.B}$ $[g]$	$x_{Comp.A}$ $\left[\frac{mol}{mol}\right]$	$x_{Comp.B}$ $\left[\frac{mol}{mol}\right]$	$T_{solidification}$ $[°C]$
0.5222	4.5540	0.10	0.90	26
1.0329	4.0579	0.20	0.80	15
1.5495	3.5519	0.30	0.70	3
2.0711	3.0482	0.40	0.60	-13
2.5827	2.5373	0.50	0.50	n.a.
3.1132	2.0384	0.60	0.40	n.a.
3.6294	1.5258	0.70	0.30	n.a.
4.1609	1.0230	0.80	0.20	-13
4.6969	0.5127	0.90	0.10	1

Table S9. Weighed masses and calculated molar compositions of components A and B and determined solidification temperature for the system FE–TH. Solidification temperatures not determined are marked with "n.a.".

$m_{Comp.A}$ $[g]$	$m_{Comp.B}$ $[g]$	$x_{Comp.A}$ $\left[\frac{mol}{mol}\right]$	$x_{Comp.B}$ $\left[\frac{mol}{mol}\right]$	$T_{solidification}$ $[°C]$
0.5285	4.5274	0.10	0.90	45
1.0654	4.0208	0.20	0.80	13
1.5879	3.5038	0.30	0.70	n.a.
2.1179	3.0002	0.40	0.60	n.a.
2.6379	2.5030	0.50	0.50	n.a.
3.1535	1.9980	0.60	0.40	n.a.
3.6758	1.4964	0.70	0.30	-26
4.1905	0.9908	0.80	0.20	-7
4.7072	0.4957	0.90	0.10	-5

1.3 Data for Ideal SLE calculations

Table S10. Solidification(=melting) temperature of pure components from SLE experiments, heat of fusion calculated with Joback [1]. Solidification temperatures from literature are taken from [2].

Pure component	$T_{solidification}$ $[°C]$	$T_{solidification,literature}$ $[°C]$	$\Delta h^{\circ}_{fusion}$ $\left[\frac{kJ}{mol}\right]$	Ideal gas constant R $\left[\frac{kJ}{mol*K}\right]$
**Camphene**	32	52	9.44	
**Fenchol**	19	41.5	9.46	
**Dodecanoic acid**	44	43.82	36.98	
**Dodecanol**	22	24.2	30.92	0.008315
**Menthol**	35	42.1	16.20	
**Thymol**	50	49.6	17.57	

2 Data for solvent extraction of aldehydes

2.1 Solvent synthesis data for solvent extraction

Table S11. Masses and compositions for synthesis of solvents used in solvent extraction.

Solvent	$m_{Comp.A}$ $[g]$	$m_{Comp.B}$ $[g]$	$x_{Comp.A}$ $[g]$	$x_{Comp.B}$ $[g]$
**CA:DA**	100.30	25.28	0.85	0.15
**CA:DO**	79.62	45.19	0.70	0.30
**CA:DO***	41.41	81.36	0.40	0.60
**CA:FE**	53.09	74.43	0.44	0.56
**CA:FE***	98.56	27.30	0.80	0.20
**FE:DA**	94.23	30.08	0.80	0.20
**FE:DA***	80.43	43.82	0.70	0.30
**FE:DO**	98.82	28.37	0.80	0.20
**FE:DO***	45.00	78.93	0.40	0.60
**CA:ME**	71.55	52.54	0.60	0.40
**CA:ME***	46.57	71.60	0.42	0.58
**CA:TH**	72.77	51.27	0.60	0.40
**CA:TH***	99.11	26.11	0.80	0.20
**TH:ME**	59.41	61.59	0.50	0.50

2.2 Water uptake during solvent extraction

Table S12. Water content of extracts after solvent extraction measured in wt.% from Karl Fischer titration and calculated into vol.%.

Solvent	$wt.\%_{water,E}$ [%]			Mean $wt.\%_{water,E}$ [%]	$vol.\%_{water,E}$ [%]	Mean $vol.\%_{water,E}$ [%]	STD $vol.\%_{water,E}$ [%]
**CA:DA**	0.108	0.058	0.057	0.074	0.065	0.061	0.01
	0.057	0.071	0.070	0.066	0.057		
**CA:DO**	0.512	0.530	0.518	0.520	0.444	0.447	0.00
	0.524	0.529	0.527	0.527	0.450		
**CA:DO***	1.469	1.421	1.461	1.450	1.226	1.220	0.01
	1.426	1.452	1.430	1.436	1.214		
**CA:FE**	1.027	1.030	1.037	1.031	0.945	0.958	0.02
	1.034	1.067	1.073	1.058	0.970		
**CA:FE***	0.209	0.231	0.231	0.224	0.198	0.200	0.00
	0.230	0.225	0.232	0.229	0.202		
**FE:DA**	2.050	2.049	2.063	2.054	1.934	1.933	0.00
	2.069	2.026	2.057	2.051	1.931		
**FE:DA***	1.943	1.911	1.926	1.927	1.798	1.815	0.02
	1.942	1.971	1.973	1.962	1.831		
**FE:DO**	2.390	2.376	2.386	2.384	2.209	2.215	0.01
	2.394	2.379	2.414	2.396	2.220		
**FE:DO***	2.332	2.361	2.329	2.341	2.051	2.044	0.01
	2.331	2.323	2.322	2.325	2.037		
**CA:ME**	0.512	0.506	0.494	0.504	0.443	0.447	0.00
	0.520	0.511	0.504	0.512	0.450		
**CA:ME***	0.871	0.870	0.835	0.859	0.760	0.757	0.00
	0.854	0.851	0.851	0.852	0.754		
**CA:TH**	0.807	0.821	0.811	0.813	0.739	0.737	0.00
	0.818	0.804	0.806	0.809	0.735		
**CA:TH***	0.310	0.298	0.302	0.303	0.269	0.271	0.00
	0.307	0.307	0.311	0.308	0.274		
**TH:ME**	1.798	1.796	1.787	1.794	1.680	1.661	0.03
	1.712	1.780	1.771	1.754	1.643		

2.3 Solvent loss during solvent extraction

Table S13. Measured Total Organic Carbon (TOC) and calculated vol.% of solvent loss.

HDES	$m_{raffinate}$ $[g]$	$m_{ultrapurewater}$ $[g]$	TOC_{meas} $[mg/L]$	$vol.\%_{solvent,R}$ [%]	Mean $vol.\%_{solvent,R}$ [%]	STD $vol.\%_{solvent,R}$ [%]
**CA:DA**	2.8164	17.5555	3.21	0.003	0.00	0.00
	5.2047	15.2851	6.13	0.003		
**CA:DO**	5.3554	15.7966	7.69	0.004	0.00	0.00
	4.5108	17.7449	7.34	0.005		
**CA:DO***	2.6295	17.6157	8.10	0.009	0.01	0.00
	3.0117	17.3357	6.31	0.006		
**CA:FE**	4.6945	14.9153	183.40	0.100	0.10	0.00
	3.5446	17.7124	121.30	0.098		
**CA:FE***	1.1118	19.5020	28.17	0.070	0.07	0.00
	3.8911	16.7327	92.41	0.065		
**FE:DA**	3.1520	17.0296	130.80	0.106	0.11	0.01
	3.1082	16.6896	127.80	0.115		
**FE:DA***	3.7736	16.6669	123.90	0.086	0.09	0.01
	3.3315	17.9698	109.90	0.101		
**FE:DO**	2.9372	17.9296	126.90	0.116	0.12	0.00
	3.3141	17.3764	134.30	0.118		
**FE:DO***	3.4440	16.5290	70.03	0.055	0.06	0.01
	3.1637	16.4945	70.34	0.065		
**CA:ME**	4.4910	15.7313	51.19	0.031	0.03	0.01
	2.9201	17.5636	39.58	0.038		
**CA:ME***	3.0015	16.9765	46.36	0.042	0.05	0.00
	2.0841	17.8260	36.09	0.049		
**CA:TH**	5.0002	15.0775	153.80	0.081	0.08	0.00
	4.0370	15.7636	127.80	0.082		
**CA:TH***	4.1767	18.8162	88.37	0.065	0.06	0.00
	3.4807	15.6155	85.98	0.062		
**TH:ME**	4.6432	15.5206	116.40	0.064	0.07	0.00
	3.5671	16.9907	86.04	0.068		

2.4 Masses of feed and solvent for solvent extractions

Table S14. Mass of feed (model solution) and mass of solvent for solvent extractions.

Extraction with solvent	$m_{model\ solution=feed}$ $[g]$	$m_{solvent}$ $[g]$
**CA:DA**	19.7160	17.3018
	19.8267	16.8299
**CA:DO**	19.8597	16.8121
	19.3556	17.0848
**CA:DO***	19.2065	16.7999
	20.0824	16.9017
**CA:FE**	19.5440	17.8865
	19.7159	17.6102
**CA:FE***	20.2323	17.4760
	19.1827	17.5727
**FE:DA**	20.1053	18.3446
	19.5805	18.2471
**FE:DA***	19.6506	17.4141
	20.0036	17.7576
**FE:DO**	19.6150	17.7215
	19.5290	18.2534
**FE:DO***	19.8544	17.1830
	19.1828	16.6260
**CA:ME**	19.9072	16.9466
	19.1923	17.2112
**CA:ME***	19.8894	16.7307
	19.6577	17.3572
**CA:TH**	20.1115	17.4896
	19.6916	17.8360
**CA:TH***	19.7235	17.4095
	19.5070	17.8186
**TH:ME**	19.6899	18.3633
	19.5411	17.6172

2.5 HPLC-UV data

Table S15. Masses of sample (model solution=feed), ethyl vanillin standard and methanol in sample preparation for HPLC analysis. Peak Areas from HPLC of ethyl vanillin (ETHVAN), p-Hydroxybenzaldehyde (HBA), vanillin (VAN), and syringaldehyde (SYR). The ID is linked to the ID of the model solution HPLC-data (Table S 16). Areas marked with 0 showed no peak for the respective aldehyde e.g. theoretical 100% extraction.

Extraction with solvent	ID	m_{sample} $[g]$	$m_{Stand.}$ $[g]$	m_{MeOH} $[g]$	A_{ETHVAN} [ ]	A_{HBA} [ ]	A_{VAN} [ ]	A_{SYR} [ ]
**CA:DA**	40423	1.0031	0.7776	2.3277	2 638 523	5 968 906	1 576 075	479 993
	40423	1.0017	0.7767	2.3188	2 648 421	5 977 671	1 595 736	479 743
**CA:DO**	40423	1.0000	0.7756	2.2992	2 671 125	1 235 847	612 362	225 116
	40423	1.0023	0.7794	2.3384	2 643 243	1 190 642	590 602	216 455
**CA:DO***	120423	0.9986	0.7344	2.2609	2 501 707	648 826	349 889	129 615
	120423	0.9873	0.7741	2.2621	2 604 143	659 465	366 692	139 733
**CA:FE**	250423	0.9944	0.7081	2.2756	2 508 499	956 757	337 917	107 693
	250423	0.9957	0.7250	2.2795	2 447 119	950 118	335 143	104 649
**CA:FE***	270423	0.9947	0.7413	2.2150	2 677 845	3 101 753	876 833	279 418
	270423	1.0040	0.7778	2.2389	2 768 831	2 978 407	837 202	278 147
**FE:DA**	250423	0.9969	0.7614	2.2711	2 695 727	813 268	299 903	93 990
	250423	0.9730	0.7374	2.2523	2 649 492	794 072	296 658	89 279
**FE:DA***	270423	1.0043	0.7660	2.2564	2 736 389	1 058 786	379 108	112 702
	270423	0.9897	0.7638	2.1962	2 762 063	1 037 952	362 004	104 775
**FE:DO**	250423	0.9905	0.7354	2.1993	2 711 362	501 694	228 721	72 358
	250423	0.9887	0.7684	2.2771	2 729 741	477 779	216 350	66 729
**FE:DO***	270423	0.9662	0.7324	2.1954	2 548 451	480 354	259 370	90 458
	270423	0.9614	0.7396	2.2633	2 639 170	503 616	271 061	91 800
**CA:ME**	120423	0.9945	0.7379	2.2556	2 506 551	1 038 405	553 326	212 215
	120423	0.9960	0.7361	2.1906	2 680 536	1 066 389	565 610	215 736
**CA:ME***	120423	0.9933	0.7716	2.2685	2 703 186	646 617	382 010	150 583
	120423	0.9740	0.7654	2.2691	2 717 815	660 615	393 077	156 620
**CA:TH**	40423	0.9863	0.7769	2.3384	2 625 460	1 809 688	120 312	25 662
	40423	1.0020	0.7769	2.3215	2 693 269	1 861 578	159 159	**0**
**CA:TH***	160523	0.9864	0.7252	2.2855	2 481 231	3 695 904	282 755	47 924
	160523	1.0043	0.7410	2.2549	2 654 774	3 895 031	296 540	52 012
**TH:ME**	160523	1.0015	0.7551	2.2918	2 554 095	470 995	103 805	21 614
	220523	1.0037	0.7537	2.2068	2 794 460	536 940	112 547	20 937

Table S16. Masses of sample (model solution=feed), ethyl vanillin standard and methanol in sample preparation for HPLC analysis. Peak Areas from HPLC of ethyl vanillin (ETHVAN), p-Hydroxybenzaldehyde (HBA), vanillin (VAN), and syringaldehyde (SYR). The ID is linked to ID of solvent extraction HPLC-data (Table S 15).

ID	m_{sample} $[g]$	$m_{Stand.}$ $[g]$	m_{MeOH} $[g]$	A_{ETHVAN} [ ]	A_{HBA} [ ]	A_{VAN} [ ]	A_{SYR} [ ]
40423	1.0080	0.7035	2.3378	2 357 684	6 473 877	2 822 153	635 929
	1.0045	0.7818	2.2922	2 668 173	6 534 793	2 857 228	662 115
120423	1.0029	0.7247	2.2659	2 476 905	6 480 702	2 830 892	657 046
	0.9905	0.7358	2.2641	2 630 245	6 734 359	2 942 372	680 179
250423	0.9968	0.7301	2.2800	2 597 250	6 716 511	2 922 026	648 988
	0.9988	0.7401	2.3136	2 476 331	6 700 892	2 924 944	659 345
270423	1.0042	0.7424	2.1752	2 617 798	6 761 187	2 953 813	677 221
	0.9962	0.7437	2.1793	2 692 976	6 871 049	3 003 335	684 760
160523	0.9599	0.7049	2.2666	2 456 372	6 334 550	2 764 451	626 821
	1.0043	0.7608	2.0957	2 683 264	6 732 154	2 931 760	658 221
220523	1.0010	0.7293	2.3309	2 597 077	6 773 887	2 945 005	673 308
	0.9964	0.7686	2.3136	2 620 244	6 519 682	2 851 491	655 284

Table S17. Calibration for HPLC from [3] here stated as rounded to zero digits after the comma.

Component	k $[g]$	d $[g]$
**Ethyl vanillin**	12878361	30416
**Vanillin**	13510093	16694
**p-Hydroxybenzaldehyde**	32103700	-4678
**Syringaldehyde**	3106900	5381

3 Material-and solvent-properties

Table S18. Purity and Molecular weight (Mw), and Number of Carbon atoms needed in TOC calculations. In case of racemic mixtures or mixtures of isomers, the structure of one enantiomer/ isomer is shown.

Component	CAS	$Purity$ [ ]	Mw $[g/mol]$	$Source$	Structure	$\#Carbon\ atoms$ [ ]
**Camphene**	79-92-5 (racemate)	0.95	136.24	Specification data sheet of supplier / Packaging of supplier	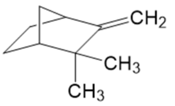	10
**Fenchol**	1632-73-1 (isomeric mixture)	0.96	154.25		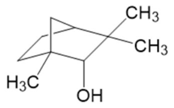	10
**Dodecanoic acid**	143-07-7	0.98	200.32		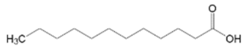	12
**Dodecanol**	112-53-8	0.98	186.33		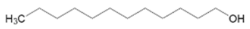	12
**Menthol**	2216-51-5 (L-Menthol)	0.99	156.27		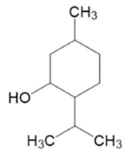	10
**Thymol**	89-83-8	0.99	150.22		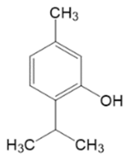	10
**Vanillin**	121-33-5	Not less than 99.5%			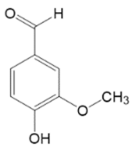	Not needed
**p-Hydroxybenzaldehyde**	123-08-0	≥98%	122,12		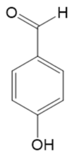	Not needed
**Syringaldehyde**	134-96-3	≥98%	182,17		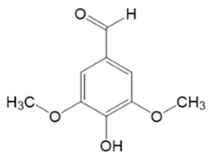	Not needed

* Computed value

Table S19. Density data measured with the Anton Paar SVM 3000 at 25°C. Density for methanol and water from respective literature.

**Component**	ρ_1 $\left[\frac{g}{cm^3}\right]$	ρ_2 $\left[\frac{g}{cm^3}\right]$	ρ_3 $\left[\frac{g}{cm^3}\right]$	ρ_4 $\left[\frac{g}{cm^3}\right]$	ρ_{mean} $\left[\frac{g}{cm^3}\right]$	**STD** $\left[\frac{g}{cm^3}\right]$	**Source**
Model solution	1.0064	1.0063	1.0044	1.0062	1.0058	0.0010	Anton Paar SVM
TOC-Model solution	1.0059	1.0056	1.0053		1.0056	0.0003	Anton Paar SVM
Methanol					0.7862		[3]
Water					0.9970		[2]
CA:DA	0.8682	0.8680	0.8680		0.8681	0.0001	Anton Paar SVM
CA:DO	0.8501	0.8500	0.8511		0.8504	0.0006	
CA:DO*	0.8397	0.8408	0.8408		0.8404	0.0006	
CA:ME	0.8766	0.8765	0.8761		0.8764	0.0003	
CA:ME*	0.8815	0.8815	0.8813		0.8814	0.0001	
CA:TH	0.9048	0.9053	0.9050		0.9050	0.0003	
CA:TH*	0.8834	0.8846	0.8845		0.8842	0.0007	
CA:FE	0.9132	0.9132	0.9132		0.9132	0.0000	
CA:FE*	0.8818	0.8819	0.8813		0.8817	0.0003	
FE:DA	0.9367	0.9367	0.9377		0.9370	0.0006	
FE:DA*	0.9290	0.9292	0.9292		0.9291	0.0001	
FE:DO	0.9235	0.9223	0.9222		0.9227	0.0007	
FE:DO*	0.8714	0.8710	0.8709		0.8711	0.0003	
TH:ME	0.9326	0.9326	0.9326		0.9326	0.0000	

Table S20. Dynamic viscosity data measured with the Anton Paar SVM 3000 at 25°C.

Component	η_1 $[mPas]$	η_2 $[mPas]$	η_3 $[mPas]$	η_{mean} $[mPas]$	STD $[mPas]$	Source
CA:DA	3.1395	3.1293	3.1383	3.1357	0.0056	Anton Paar SVM
CA:DO	4.4290	4.4576	4.4752	4.4539	0.0233	
CA:DO*	8.4548	8.5156	8.4561	8.4755	0.0347	
CA:ME	4.8983	4.9031	4.8816	4.8943	0.0113	
CA:ME*	9.0291	8.9094	8.8947	8.9444	0.0737	
CA:TH	3.4333	3.4372	3.4369	3.4358	0.0022	
CA:TH*	2.5142	2.5263	2.5367	2.5257	0.0113	
CA:FE	8.5146	8.4914	8.3878	8.4646	0.0675	
CA:FE*	3.0894	3.0537	3.0581	3.0671	0.0195	
FE:DA	34.1710	34.1250	35.5560	34.6173	0.8132	
FE:DA*	29.8570	29.0800	29.6050	29.5140	0.3964	
FE:DO	32.4050	31.4040	31.5940	31.8010	0.5316	
FE:DO*	21.2610	21.1180	21.0140	21.1310	0.1240	
TH:ME	34.4010	35.0360	34.7860	34.7410	0.3199	

Table S21. Kinematic viscosity data measured with the Anton Paar SVM 3000 at 25°C.

Component	υ_1 $\left[\frac{mm^2}{s}\right]$	υ_2 $\left[\frac{mm^2}{s}\right]$	υ_2 $\left[\frac{mm^2}{s}\right]$	υ_{mean} $\left[\frac{mm^2}{s}\right]$	STD $\left[\frac{mm^2}{s}\right]$	Source
CA:DA	3.6162	3.6052	3.6156	3.6123	0.0062	Anton Paar SVM
CA:DO	5.2102	5.2444	5.2584	5.2377	0.0248	
CA:DO*	10.0690	10.1290	10.0570	10.0850	0.0386	
CA:ME	5.5876	5.5937	5.5722	5.5845	0.0111	
CA:ME*	10.2430	10.1070	10.0920	10.1473	0.0832	
CA:TH	3.7943	3.7968	3.7977	3.7963	0.0018	
CA:TH*	2.8462	2.8858	2.8679	2.8666	0.0198	
CA:FE	9.3237	9.2987	9.1849	9.2691	0.0740	
CA:FE*	3.5037	3.4628	3.4704	3.4790	0.0218	
FE:DA	36.4810	36.4300	37.9200	36.9437	0.8459	
FE:DA*	32.1370	31.2970	31.8610	31.7650	0.4281	
FE:DO	35.0900	34.0490	34.2580	34.4657	0.5507	
FE:DO*	24.3980	24.2450	24.1280	24.2570	0.1354	
TH:ME	36.8870	37.5690	37.2990	37.2517	0.3435	

4 GC-Data

Table S22. GC-Areas of component peaks before and after the TOC extraction.

Solvent (A:B)	Before extraction		After extraction	
	GC-Area Component A	GC-Area Component B	GC-Area Component A	GC-Area Component B
**TH:ME**	21088	21319	22374	22557
**CA:DA**	15978	1090	16284	1139
**CA:DO**	12534	4552	12580	4532
**CA:DO***	67957	86086	44386	55301
**CA:FE**	64787	65610	42890	42505
**CA:FE***	77863	15003	75575	14473
**FE:DA**	48284	6990	51439	7831
**FE:DA***	32411	8457	37218	10628
**FE:DO**	49756	11622	48680	11365
**FE:DO***	22152	31429	29337	41773
**CA:ME**	206546	115261	159073	84103
**CA:ME***	125797	165065	98204	122147
**CA:TH**	13004	7235	12723	7067
**CA:TH***	60592	11503	61306	11884

5 ^{1}H-NMR spectra of FE:DO and FE:DA

Table S23. Masses and composition of solvents FE:DA and FE:DO analyzed by ^{1}H-NMR.

Solvents	$m_{Comp.A}$ $[g]$	$m_{Comp.B}$ $[g]$	$x_{Comp.A}$ $\left[\frac{mol}{mol}\right]$	$x_{Comp.B}$ $\left[\frac{mol}{mol}\right]$
FE:DA	3.9113	1.2589	0.80	0.20
FE:DO	4.0296	1.2012	0.80	0.20

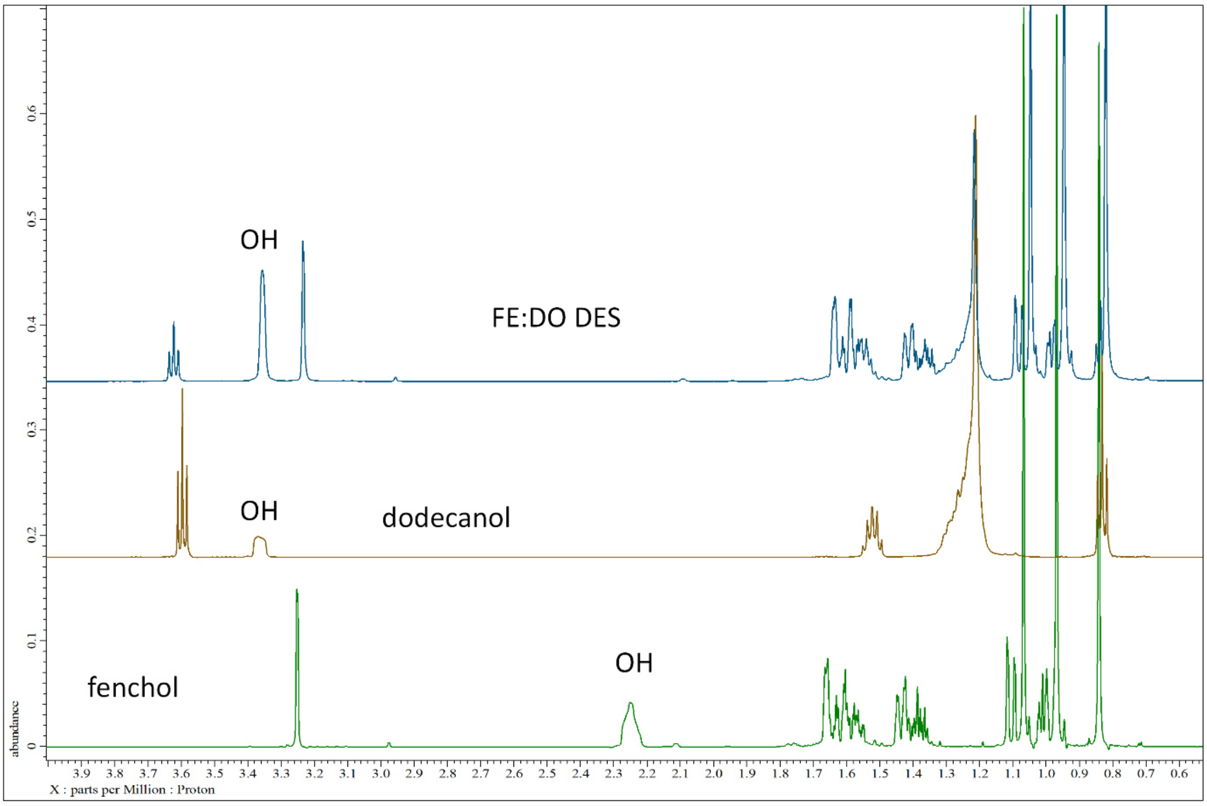

Figure S1. ^{1}H-NMR spectra in $CDCl_3$ of fenchol, dodecanol and FE:DO DES at 30 °C. In FE:DO DES the OH-signals of both components have the same chemical shift, which is a strong indication of hydrogen bonding between the two partners.

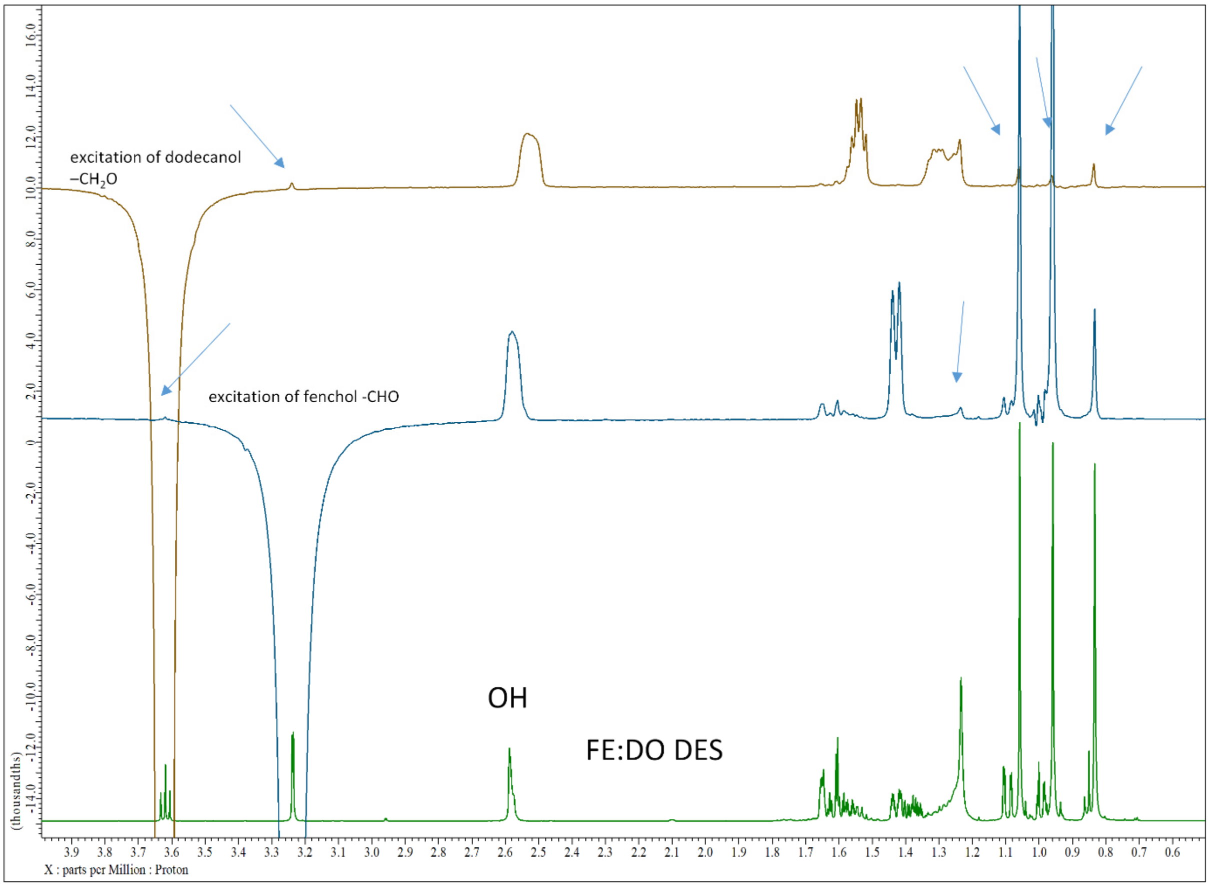

Figure S2. ^{1}H-NMR and NOESY1D-NMR spectra in $CDCl_3$ of FE:DO DES at 35 °C. The higher temperature was chosen to shift the OH-resonance out of the crowded regions. Excitation of the low-field signals of both partners gives also intermolecular NOEs (arrows). Therefore, the two partners have to be in close proximity.

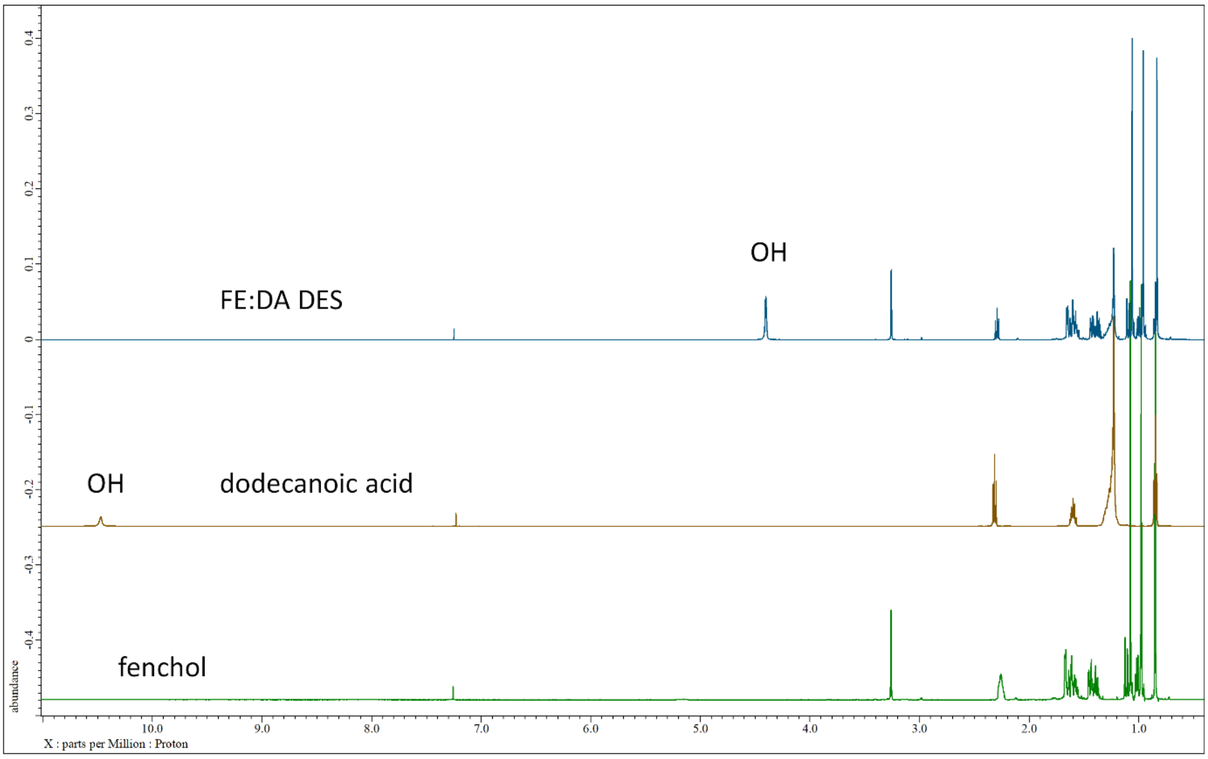

Figure S3. ^{1}H-NMR spectra in $CDCl_3$ of fenchol, dodecanoic acid and FE:DA DES at 30 °C. In FE:DA DES the OH-signals of both components have the same chemical shift, which is a strong indication of hydrogen bonding between the two partners.

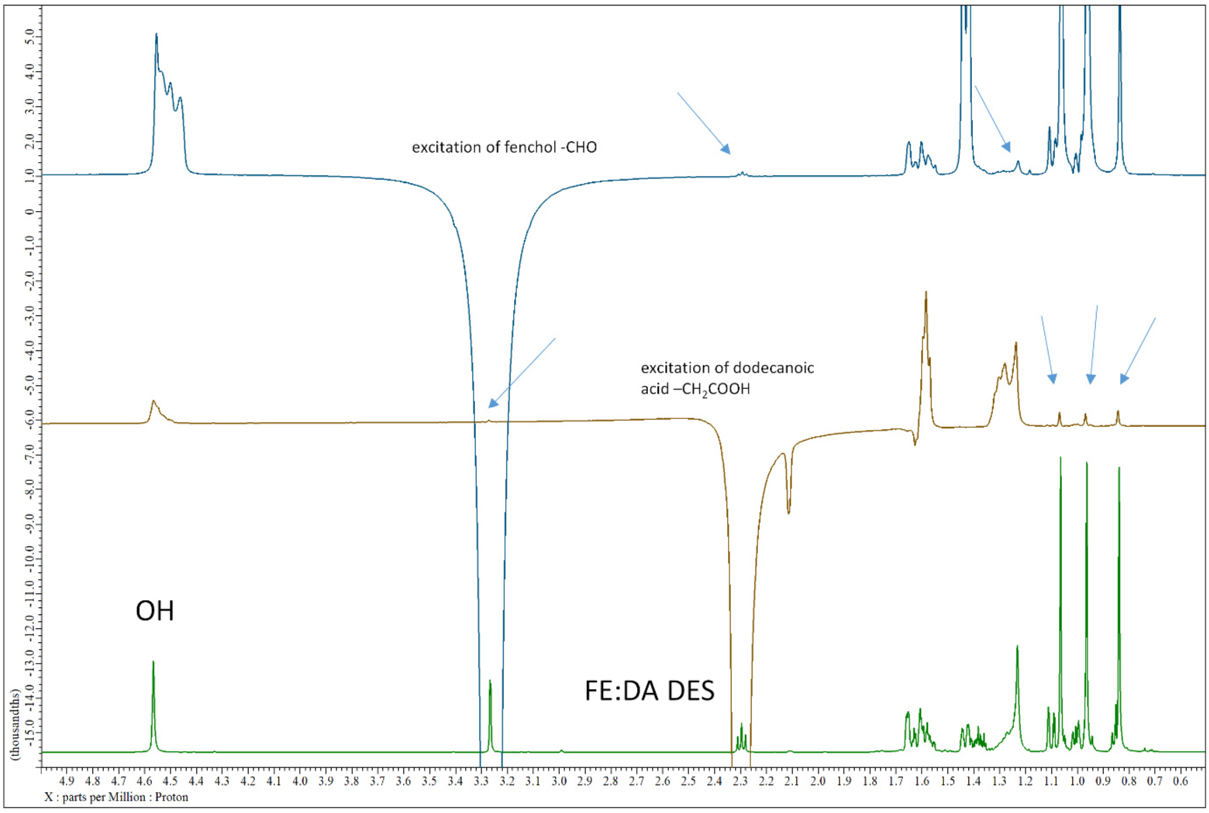

Figure S4. ^{1}H-NMR and NOESY1D-NMR spectra in $CDCl_3$ of FE:DA DES at 30 °C. Excitation of the low-field signals of both partners gives also intermolecular NOEs (arrows). Therefore, the two partners have to be in close proximity.

6 References

1. JOBACK, K.G.; REID, R.C. ESTIMATION OF PURE-COMPONENT PROPERTIES FROM GROUP-CONTRIBUTIONS. *Chemical Engineering Communications* **1987**, *57*, 233–243, doi:10.1080/00986448708960487.
2. *CRC Handbook of chemistry and physics;* Haynes, W.M., Ed., 97th edition; CRC Press, 2017, ISBN 9781498754286.
3. Kaufmann, A.; Maier, L.; Kienberger, M. Solvent screening for the extraction of aromatic aldehydes. *Separation and Purification Technology* **2024**, *340*, 126780, doi:10.1016/j.seppur.2024.126780.
